# Supplementary material for: Individual lipid alterations at the origin of neuronal Ceramide Synthase defects
Source: PLoS Genet. 2025 Sep 25;21(9):e1011880. doi: 10.1371/journal.pgen.1011880 (PMC12500085; doi:10.1371/journal.pgen.1011880)
Supplement: S1 Table — Genotypes used. (DOCX) [file pgen.1011880.s006.docx]

Genotypes used:

Figure 1B-F

| Name | Genotype |
| --- | --- |
| *cerS^WT^* | P{ry[+t7.2]=hsFLP}1, P{w[+mC]=tubP-GAL80}LL1 w[*] P{ry[+t7.2]=neoFRT}19A/*cerS^WT^*, P{ry[+t7.2]=neoFRT}19A; P{w[+mW.hs]=GawB}109(2)80, P{w[+mC]=UAS-mCD8::GFP.L}LL5 P{w[+mC]=SOP-FLP}73 / + |
| *cerS^KO^* | P{ry[+t7.2]=hsFLP}1, P{w[+mC]=tubP-GAL80}LL1 w[*] P{ry[+t7.2]=neoFRT}19A/*cerS^KO^*, P{ry[+t7.2]=neoFRT}19A; P{w[+mW.hs]=GawB}109(2)80, P{w[+mC]=UAS-mCD8::GFP.L}LL5 P{w[+mC]=SOP-FLP}73 / + |
| *cerS^H215D^* | P{ry[+t7.2]=hsFLP}1, P{w[+mC]=tubP-GAL80}LL1 w[*] P{ry[+t7.2]=neoFRT}19A/*cerS^H215D^*, P{ry[+t7.2]=neoFRT}19A; P{w[+mW.hs]=GawB}109(2)80, P{w[+mC]=UAS-mCD8::GFP.L}LL5 P{w[+mC]=SOP-FLP}73 / + |
| rescue | P{ry[+t7.2]=hsFLP}1, P{w[+mC]=tubP-GAL80}LL1 w[*] P{ry[+t7.2]=neoFRT}19A/*cerS^KO^*, P{ry[+t7.2]=neoFRT}19A; P{w[+mW.hs]=GawB}109(2)80, P{w[+mC]=UAS-mCD8::GFP.L}LL5 P{w[+mC]=SOP-FLP}73 / +; UAS-cerS |

Figure 1G

| Name | Genotype |
| --- | --- |
| UAS-cerS^dsRNA1^/+ | w; *UAS-cerS^dsRNA1^*/+ |
| 21-7-Gal4>UAS-Dicer2/+ | w; P{w[+mC]=GAL4}21-7, *UAS -Dicer2*/+; + / *ppk CD4 tdTomato* |
| 21-7-Gal4>UAS-Dicer2/+ | w; P{w[+mC]=GAL4}21-7, *UAS -Dicer2*/ *UAS-cerS^dsRNA1^*; + / *ppk CD4 tdTomato* |
| UAS-cerS^dsRNA2^/+ | w; +; *UAS-cerS^dsRNA2^*/+ |
| 21-7-Gal4>UAS-Dicer2/+ | w; P{w[+mC]=GAL4}21-7, *UAS -Dicer2*/+; + / *ppk CD4 tdTomato* |
| 21-7-Gal4>UAS-Dicer2/UAS- cerS^dsRNA2^ | w; P{w[+mC]=GAL4}21-7, *UAS -Dicer2*/+; *UAS-cerS^dsRNA2^* / *ppk CD4 tdTomato* |

Figure S1

| Name | Genotype |
| --- | --- |
| *cerS^WT^* | P{ry[+t7.2]=hsFLP}1, P{w[+mC]=tubP-GAL80}LL1 w[*] P{ry[+t7.2]=neoFRT}19A/*cerS^WT^*, P{ry[+t7.2]=neoFRT}19A; P{w[+mW.hs]=GawB}109(2)80, P{w[+mC]=UAS-mCD8::GFP.L}LL5 P{w[+mC]=SOP-FLP}73 / + |
| *cerS^KO^* | P{ry[+t7.2]=hsFLP}1, P{w[+mC]=tubP-GAL80}LL1 w[*] P{ry[+t7.2]=neoFRT}19A/*cerS^KO^*, P{ry[+t7.2]=neoFRT}19A; P{w[+mW.hs]=GawB}109(2)80, P{w[+mC]=UAS-mCD8::GFP.L}LL5 P{w[+mC]=SOP-FLP}73 / + |
| *rescue* | P{ry[+t7.2]=hsFLP}1, P{w[+mC]=tubP-GAL80}LL1 w[*] P{ry[+t7.2]=neoFRT}19A/*cerS^KO^*, P{ry[+t7.2]=neoFRT}19A; P{w[+mW.hs]=GawB}109(2)80, P{w[+mC]=UAS-mCD8::GFP.L}LL5 P{w[+mC]=SOP-FLP}73 / +; UAS-cerS |
| *cerS^KO^*/+ | *cerS^KO^*, P{ry[+t7.2]=neoFRT}19A/+ ;; *ppk-mCD8GFP/+* |

Figure S2

| Name | Genotype |
| --- | --- |
| *cerS^KO^* | P{ry[+t7.2]=hsFLP}1, P{w[+mC]=tubP-GAL80}LL1 w[*] P{ry[+t7.2]=neoFRT}19A/*cerS^KO^*, P{ry[+t7.2]=neoFRT}19A; P{w[+mW.hs]=GawB}109(2)80, P{w[+mC]=UAS-mCD8::GFP.L}LL5 P{w[+mC]=SOP-FLP}73 / + |
| *cerS^KO^; UAS-p53* | P{ry[+t7.2]=hsFLP}1, P{w[+mC]=tubP-GAL80}LL1 w[*] P{ry[+t7.2]=neoFRT}19A/*cerS^KO^*, P{ry[+t7.2]=neoFRT}19A; P{w[+mW.hs]=GawB}109(2)80, P{w[+mC]=UAS-mCD8::GFP.L}LL5 P{w[+mC]=SOP-FLP}73 / UAS-p35 |
| *cerS^KO^;+;UAS-p53* | P{ry[+t7.2]=hsFLP}1, P{w[+mC]=tubP-GAL80}LL1 w[*] P{ry[+t7.2]=neoFRT}19A/*cerS^KO^*, P{ry[+t7.2]=neoFRT}19A; P{w[+mW.hs]=GawB}109(2)80, P{w[+mC]=UAS-mCD8::GFP.L}LL5 P{w[+mC]=SOP-FLP}73 / +; UAS-p35/+ |
| *ppk-Gal4 > UAS-cerS^dsRNA1^* | +/+; *UAS-cerS^dsRNA1^/UAS-dicer2; ppk-Gal4, UAS-mCD8GFP/+* |
| wildtype | +/+; *+/UAS-dicer2; ppk-Gal4, UAS-mCD8GFP/+* |

Figure 2A

| Name | Genotype |
| --- | --- |
| control | *white^1118^;* ppk-CD4tdTomato |
| *cerS^P61^* | *white^67c23^*, P{lacW}schlankG0061; ppk-CD4tdTomato |

Figure 2B/D

| Name | Genotype |
| --- | --- |
| *white^1118^* | *white^1118^* |
| *cerS^P61^* | *white^67c23^*, P{lacW}schlankG0061/FM7c |

Figure 3

| Name | Genotype |
| --- | --- |
| *cerS^WT^* | P{ry[+t7.2]=hsFLP}1, P{w[+mC]=tubP-GAL80}LL1 w[*] P{ry[+t7.2]=neoFRT}19A/*cerS^WT^*, P{ry[+t7.2]=neoFRT}19A; P{w[+mW.hs]=GawB}109(2)80, P{w[+mC]=UAS-mCD8::GFP.L}LL5 P{w[+mC]=SOP-FLP}73 / + |
| *FRT40A* | P{w[+m*]=GAL4}5-40 P{w[+mC]=UAS-Venus.pm}1 P{w[+mC]=SOP-FLP}42/+; P{w[+mC]=tubP-GAL80}LL10 P{ry[+t7.2]=neoFRT}40A / P{ry[+t7.2]=neoFRT}40A |
| *lace^SK6^, FRT40A* | P{w[+m*]=GAL4}5-40 P{w[+mC]=UAS-Venus.pm}1 P{w[+mC]=SOP-FLP}42/+; P{w[+mC]=tubP-GAL80}LL10 P{ry[+t7.2]=neoFRT}40A / *laceSK6,* P{ry[+t7.2]=neoFRT}40A |
| *cerS^H215D^* | P{ry[+t7.2]=hsFLP}1, P{w[+mC]=tubP-GAL80}LL1 w[*] P{ry[+t7.2]=neoFRT}19A/*cerS^H215D^*, P{ry[+t7.2]=neoFRT}19A; P{w[+mW.hs]=GawB}109(2)80, P{w[+mC]=UAS-mCD8::GFP.L}LL5 P{w[+mC]=SOP-FLP}73 / + |
| *des1^KO^, FRT40A* | P{w[+m*]=GAL4}5-40 P{w[+mC]=UAS-Venus.pm}1 P{w[+mC]=SOP-FLP}42/+; P{w[+mC]=tubP-GAL80}LL10 P{ry[+t7.2]=neoFRT}40A / *des1^KO^,* P{ry[+t7.2]=neoFRT}40A |
| *FRT42D* | P{w[+m*]=GAL4}5-40 P{w[+mC]=UAS-Venus.pm}1 P{w[+mC]=SOP-FLP}42/+; P{ry[+t7.2]=neoFRT}42D P{w[+mC]=tubP-GAL80}LL2 / P{ry[+t7.2]=neoFRT}42D |
| *cpes^KO^, FRT42D* | P{w[+m*]=GAL4}5-40 P{w[+mC]=UAS-Venus.pm}1 P{w[+mC]=SOP-FLP}42/+; P{ry[+t7.2]=neoFRT}42D P{w[+mC]=tubP-GAL80}LL2 / P{ry[+t7.2]=neoFRT}42D, *cpes^KO^* |

Figure S3

| Name | Genotype |
| --- | --- |
| *control^dsRNA^* | *UAS-Dicer2/UAS-OR42B^dsRNA^; ppk-Gal4, UAS-mCD8GFP/+* |
| *UAS-spt1^dsRNA1^* | *UAS-Dicer2/UAS-spt1^dsRNA1^; ppk-Gal4, UAS-mCD8GFP/+* |
| *UAS-spt1^dsRNA2^* | *UAS-Dicer2/UAS-spt1^dsRNA2^; ppk-Gal4, UAS-mCD8GFP/+* |
| *UAS-lace^dsRNA1^* | *UAS-Dicer2/+; ppk-Gal4, UAS-mCD8GFP/ UAS-lace^dsRNA1^* |
| *UAS-lace^dsRNA2^* | *UAS-Dicer2/UAS-lace^dsRNA1^; ppk-Gal4, UAS-mCD8GFP/+* |
| *UAS-aKR^dsRNA1^* | *UAS-Dicer2/+; ppk-Gal4, UAS-mCD8GFP/ UAS-aKR^dsRNA1^* |
| *UAS-aKR^dsRNA2^* | *UAS-Dicer2/UAS-aKR^dsRNA2^; ppk-Gal4, UAS-mCD8GFP/+* |
| *UAS-cerS^dsRNA1^* | *UAS-Dicer2/UAS-cerS^dsRNA1^; ppk-Gal4, UAS-mCD8GFP/+* |
| *UAS-cerS^dsRNA2^* | *UAS-Dicer2/+; ppk-Gal4, UAS-mCD8GFP/ UAS-cerS^dsRNA2^* |
| *UAS-des1^dsRNA1^* | *UAS-Dicer2/UAS-des1^dsRNA1^; ppk-Gal4, UAS-mCD8GFP/+* |
| *UAS-cpeS^dsRNA1^* | *UAS-Dicer2/+; ppk-Gal4, UAS-mCD8GFP/ UAS-cpeS^dsRNA1^* |
| *UAS-cpeS^dsRNA2^* | *UAS-Dicer2/UAS-cpeS^dsRNA2^; ppk-Gal4, UAS-mCD8GFP/+* |
| *UAS-glcT^dsRNA1^* | *UAS-Dicer2/UAS-glcT^dsRNA1^; ppk-Gal4, UAS-mCD8GFP/+* |
| *UAS-glcT^dsRNA2^* | *UAS-Dicer2/UAS-glcT^dsRNA2^; ppk-Gal4, UAS-mCD8GFP/+* |

Figure S3

| Name | Genotype |
| --- | --- |
| control | *UAS-Dicer2/UAS-OR42B^dsRNA^; ppk-Gal4, UAS-mCD8GFP/+* |
| *ppk-Gal4 > cerS^dsRNA1^* | *UAS-Dicer2/UAS-cerS^dsRNA1^; ppk-Gal4, UAS-mCD8GFP/+* |
| *ppk-Gal4 > cerS^dsRNA2^* | *UAS-Dicer2/+; ppk-Gal4, UAS-mCD8GFP/ UAS-cerS^dsRNA2^* |
| *A58-Gal4 > UAS-cerS^dsRNA1^* | *UAS-Dicer2/ UAS-cerS^dsRNA1^; ppk-mCD8GFP, A58-Gal4/ +* |
| *A58-Gal4 > UAS-cerS^dsRNA2^* | *UAS-Dicer2/+; ppk-mCD8GFP, A58-Gal4/ UAS-cerS^dsRNA2^* |
| *repo-Gal4 > UAS-cerS^dsRNA1^* | *UAS-Dicer2/ UAS-cerS^dsRNA1^; ppk-mCD8GFP/ repo-Gal4* |

Figure 4B

| Name | Genotype |
| --- | --- |
| *white^1118^* | *white^1118^* |
| *cerS^P61^* | *white^67c23^*, P{lacW}schlankG0061/FM7c |

Figure 4C/D

| Name | Genotype |
| --- | --- |
| *cerS^WT^* | P{ry[+t7.2]=hsFLP}1, P{w[+mC]=tubP-GAL80}LL1 w[*] P{ry[+t7.2]=neoFRT}19A/*cerS^WT^*, P{ry[+t7.2]=neoFRT}19A; P{w[+mW.hs]=GawB}109(2)80, P{w[+mC]=UAS-mCD8::GFP.L}LL5 P{w[+mC]=SOP-FLP}73 / + |
| *cerS^WT^+ UAS-SK1* | P{ry[+t7.2]=hsFLP}1, P{w[+mC]=tubP-GAL80}LL1 w[*] P{ry[+t7.2]=neoFRT}19A/*cerS^WT^*, P{ry[+t7.2]=neoFRT}19A; P{w[+mW.hs]=GawB}109(2)80, P{w[+mC]=UAS-mCD8::GFP.L}LL5 P{w[+mC]=SOP-FLP}73 / UAS-sk1 |
| *cerS^WT^+ UAS-SK2* | P{ry[+t7.2]=hsFLP}1, P{w[+mC]=tubP-GAL80}LL1 w[*] P{ry[+t7.2]=neoFRT}19A/*cerS^WT^*, P{ry[+t7.2]=neoFRT}19A; P{w[+mW.hs]=GawB}109(2)80, P{w[+mC]=UAS-mCD8::GFP.L}LL5 P{w[+mC]=SOP-FLP}73 / UAS-sk2 |
| *cerS^H215D^* | P{ry[+t7.2]=hsFLP}1, P{w[+mC]=tubP-GAL80}LL1 w[*] P{ry[+t7.2]=neoFRT}19A/*cerS^H215D^*, P{ry[+t7.2]=neoFRT}19A; P{w[+mW.hs]=GawB}109(2)80, P{w[+mC]=UAS-mCD8::GFP.L}LL5 P{w[+mC]=SOP-FLP}73 / + |
| *cerS^H215D^ + UAS-SK1* | P{ry[+t7.2]=hsFLP}1, P{w[+mC]=tubP-GAL80}LL1 w[*] P{ry[+t7.2]=neoFRT}19A/*cerS^H215D^*, P{ry[+t7.2]=neoFRT}19A; P{w[+mW.hs]=GawB}109(2)80, P{w[+mC]=UAS-mCD8::GFP.L}LL5 P{w[+mC]=SOP-FLP}73 / UAS-sk1 |
| *cerS^H215D^ + UAS-SK2* | P{ry[+t7.2]=hsFLP}1, P{w[+mC]=tubP-GAL80}LL1 w[*] P{ry[+t7.2]=neoFRT}19A/*cerS^H215D^*, P{ry[+t7.2]=neoFRT}19A; P{w[+mW.hs]=GawB}109(2)80, P{w[+mC]=UAS-mCD8::GFP.L}LL5 P{w[+mC]=SOP-FLP}73 / UAS-sk2 |

Figure 4E/F

| Name | Genotype |
| --- | --- |
| *cerS^H215D^* | P{ry[+t7.2]=hsFLP}1, P{w[+mC]=tubP-GAL80}LL1 w[*] P{ry[+t7.2]=neoFRT}19A/*cerS^H215D^*, P{ry[+t7.2]=neoFRT}19A; P{w[+mW.hs]=GawB}109(2)80, P{w[+mC]=UAS-mCD8::GFP.L}LL5 P{w[+mC]=SOP-FLP}73 / + |
| *cerS^H215D^ + UAS-lace^dsRNA1^* | P{ry[+t7.2]=hsFLP}1, P{w[+mC]=tubP-GAL80}LL1 w[*] P{ry[+t7.2]=neoFRT}19A/*cerS^H215D^*, P{ry[+t7.2]=neoFRT}19A; P{w[+mW.hs]=GawB}109(2)80, P{w[+mC]=UAS-mCD8::GFP.L}LL5 P{w[+mC]=SOP-FLP}73 / +; *UAS-lace^dsRNA1^*/+ |
| *cerS^H215D^+ UAS-sk2^dsRNA1^* | P{ry[+t7.2]=hsFLP}1, P{w[+mC]=tubP-GAL80}LL1 w[*] P{ry[+t7.2]=neoFRT}19A/*cerS^H215D^*, P{ry[+t7.2]=neoFRT}19A; P{w[+mW.hs]=GawB}109(2)80, P{w[+mC]=UAS-mCD8::GFP.L}LL5 P{w[+mC]=SOP-FLP}73 / *UAS-sk2^dsRNA1^* |
| *cerS^H215D^+ UAS-tre1^dsRNA1^* | P{ry[+t7.2]=hsFLP}1, P{w[+mC]=tubP-GAL80}LL1 w[*] P{ry[+t7.2]=neoFRT}19A/*cerS^H215D^*, P{ry[+t7.2]=neoFRT}19A; P{w[+mW.hs]=GawB}109(2)80, P{w[+mC]=UAS-mCD8::GFP.L}LL5 P{w[+mC]=SOP-FLP}73 / +; *UAS-tre1^dsRNA1^*/+ |
| *cerS^H215D^+ UAS-sply* | P{ry[+t7.2]=hsFLP}1, P{w[+mC]=tubP-GAL80}LL1 w[*] P{ry[+t7.2]=neoFRT}19A/*cerS^H215D^*, P{ry[+t7.2]=neoFRT}19A; P{w[+mW.hs]=GawB}109(2)80, P{w[+mC]=UAS-mCD8::GFP.L}LL5 P{w[+mC]=SOP-FLP}73 / +; UAS-slpy/+ |

Figure 5A

| Name | Genotype |
| --- | --- |
| *white^1118^* | *white^1118^* |
| *cerS^P61^* | *white^67c23^*, P{lacW}schlankG0061/FM7c |

Figure 5C/D

| Name | Genotype |
| --- | --- |
| *cerS^WT^* | P{ry[+t7.2]=hsFLP}1, P{w[+mC]=tubP-GAL80}LL1 w[*] P{ry[+t7.2]=neoFRT}19A/*cerS^WT^*, P{ry[+t7.2]=neoFRT}19A; P{w[+mW.hs]=GawB}109(2)80, P{w[+mC]=UAS-mCD8::GFP.L}LL5 P{w[+mC]=SOP-FLP}73 / + |
| *cerS^H215D^* | P{ry[+t7.2]=hsFLP}1, P{w[+mC]=tubP-GAL80}LL1 w[*] P{ry[+t7.2]=neoFRT}19A/*cerS^H215D^*, P{ry[+t7.2]=neoFRT}19A; P{w[+mW.hs]=GawB}109(2)80, P{w[+mC]=UAS-mCD8::GFP.L}LL5 P{w[+mC]=SOP-FLP}73 / + |
| *cerS^H215D^ + UAS-CerS5* | P{ry[+t7.2]=hsFLP}1, P{w[+mC]=tubP-GAL80}LL1 w[*] P{ry[+t7.2]=neoFRT}19A/*cerS^H215D^*, P{ry[+t7.2]=neoFRT}19A; P{w[+mW.hs]=GawB}109(2)80, P{w[+mC]=UAS-mCD8::GFP.L}LL5 P{w[+mC]=SOP-FLP}73 / UAS-CerS5 |
| *cerS^H215D^ + UAS-CerS1* | P{ry[+t7.2]=hsFLP}1, P{w[+mC]=tubP-GAL80}LL1 w[*] P{ry[+t7.2]=neoFRT}19A/*cerS^H215D^*, P{ry[+t7.2]=neoFRT}19A; P{w[+mW.hs]=GawB}109(2)80, P{w[+mC]=UAS-mCD8::GFP.L}LL5 P{w[+mC]=SOP-FLP}73 / UAS-CerS1 |
| *cerS^H215D^ + UAS-CerS4* | P{ry[+t7.2]=hsFLP}1, P{w[+mC]=tubP-GAL80}LL1 w[*] P{ry[+t7.2]=neoFRT}19A/*cerS^H215D^*, P{ry[+t7.2]=neoFRT}19A; P{w[+mW.hs]=GawB}109(2)80, P{w[+mC]=UAS-mCD8::GFP.L}LL5 P{w[+mC]=SOP-FLP}73 / UAS-CerS4 |
| *cerS^H215D^ + UAS-CerS2* | P{ry[+t7.2]=hsFLP}1, P{w[+mC]=tubP-GAL80}LL1 w[*] P{ry[+t7.2]=neoFRT}19A/*cerS^H215D^*, P{ry[+t7.2]=neoFRT}19A; P{w[+mW.hs]=GawB}109(2)80, P{w[+mC]=UAS-mCD8::GFP.L}LL5 P{w[+mC]=SOP-FLP}73 / +; UAS- CerS2/+ |

Figure 6A

| Name | Genotype |
| --- | --- |
| *white^1118^* | *white^1118^* |
| Tub-Gal4 > UAS-CerS5 | UAS-CerS5/+; {w[+mC]=tubP-GAL4}LL7/+ |

Figure 6B-D

| Name | Genotype |
| --- | --- |
| control | w;;ppk-Gal4, UAS-mCD8GFP/+ |
| UAS-mCerS5 | w;UAS-mCerS5/+;ppk-Gal4, UAS-mCD8GFP/+ |
| UAS-mCerS1 | w;UAS-mCerS1/+;ppk-Gal4, UAS-mCD8GFP/+ |
| UAS-mCerS4 | w;UAS-mCerS4/+;ppk-Gal4, UAS-mCD8GFP/+ |
| UAS-mCerS2 | w;;ppk-Gal4, UAS-mCD8GFP/UAS-mCerS2 |

Figure S5

| Name | Genotype |
| --- | --- |
| *cerS^H215D^* | P{ry[+t7.2]=hsFLP}1, P{w[+mC]=tubP-GAL80}LL1 w[*] P{ry[+t7.2]=neoFRT}19A/*cerS^H215D^*, P{ry[+t7.2]=neoFRT}19A; P{w[+mW.hs]=GawB}109(2)80, P{w[+mC]=UAS-mCD8::GFP.L}LL5 P{w[+mC]=SOP-FLP}73 / + |
| *cerS^WT^* | P{ry[+t7.2]=hsFLP}1, P{w[+mC]=tubP-GAL80}LL1 w[*] P{ry[+t7.2]=neoFRT}19A/*cerS^WT^*, P{ry[+t7.2]=neoFRT}19A; P{w[+mW.hs]=GawB}109(2)80, P{w[+mC]=UAS-mCD8::GFP.L}LL5 P{w[+mC]=SOP-FLP}73 / + |
