# Supplementary material for: Individual lipid alterations at the origin of neuronal Ceramide Synthase defects
Source: PLoS Genet. 2025 Sep 25;21(9):e1011880. doi: 10.1371/journal.pgen.1011880 (PMC12500085; doi:10.1371/journal.pgen.1011880)
Supplement: S2 Table — Minimal data set. (DOCX) [file pgen.1011880.s007.docx]

Minimal Dataset:

**p-values always:**

* <0.05

**<0.005

***<0.0005

****<0.00005

**FIGURE 1**

| Figure 1B |  |  |  |
| --- | --- | --- | --- |
|  |  |  |  |
| length in mm |  |  |  |
|  |  |  |  |
|  |  |  |  |
| \|  \| ***CerS^WT^*** \| ***cerS^KO^*** \| ***cerS^H215D^*** \| ***rescue*** \| \| --- \| --- \| --- \| --- \| --- \| \|  \| 17,576 \| 3,9382 \| 5,5256 \| 17,968 \| \|  \| 19,814 \| 5,4918 \| 5,2793 \| 18,691 \| \|  \| 22,16 \| 4,7183 \| 5,9755 \| 17,164 \| \|  \| 18,752 \| 5,5593 \| 4,3673 \| 13,005 \| \|  \| 23,656 \| 5,6464 \| 4,5506 \| 10,211 \| \|  \| 22,732 \| 5,7764 \| 4,5133 \| 11,66 \| \|  \| 15,032 \| 5,3473 \| 4,6941 \| 16,922 \| \| **mean** \| **19.96** \| **5.211** \| **5.976** \| **15.09** \| \| **SEM** \| **1.171** \| **0.2484** \| **0.2308** \| **1.28** \| |  |  |  |
|  |  |  |  |
| One-Way ANOVA with Tukey´s comparison test  Figure 1B  Terminals |  |  |  |
| \|  \| ***CerS^WT^*** \| ***cerS^KO^*** \| ***cerS^H215D^*** \| ***rescue*** \| \| --- \| --- \| --- \| --- \| --- \| \|  \| 780 \| 48 \| 89 \| 601 \| \|  \| 619 \| 69 \| 62 \| 687 \| \|  \| 827 \| 48 \| 68 \| 630 \| \|  \| 676 \| 91 \| 49 \| 519 \| \|  \| 683 \| 104 \| 69 \| 447 \| \|  \| 891 \| 127 \| 48 \| 401 \| \|  \| 577 \| 80 \| 43 \| 542 \| \| **mean** \| **721.9** \| **81** \| **61.14** \| **546.7** \| \| **SEM** \| **43.16** \| **10.98** \| **6.045** \| **38.28** \| |  |  |  |
|  |  |  |  |
|  |  |  |  |
| Statistical test used: One-Way ANOVA with Tukey´s comparison test  Figure 1E  length   \|  \| ***CerS^WT^*** \| ***CerS^WT^+24h*** \| \| --- \| --- \| --- \| \|  \| 13,864 \| 15,467 \| \|  \| 13,061 \| 17,716 \| \|  \| 11,378 \| 16,478 \| \|  \| 9,716 \| 15,787 \| \|  \| 13,003 \| 17,612 \| \| **mean** \| 12.2 \| 16.61 \| \| **SEM** \| 0.7416 \| 0.4598 \| |  |  |  |

Paired t-test

Terminals

|  | ***CerS^WT^*** | ***CerS^WT^+24h*** |
| --- | --- | --- |
|  | 655 | 951 |
|  | 602 | 813 |
|  | 550 | 694 |
|  | 510 | 695 |
|  | 686 | 801 |
| **mean** | 600.6 | 790.8 |
| **SEM** | 32.42 | 47.33 |

Paired t-test

Figure 1F

Length

|  | ***CerS^H215D^*** | ***Cer^H215D^+24h*** |
| --- | --- | --- |
|  | 7,7167 | 5,7896 |
|  | 6,904 | 7,1359 |
|  | 7,9424 | 8,259 |
|  | 6,1943 | 6,5156 |
|  | 6,6796 | 5,4691 |
|  | 6,4737 | 6,5237 |
| **mean** | 6.985 | 6.615 |
| **SEM** | 0.2851 | 0.4077 |

Paired t-test

Terminals

|  | ***CerS^H215D^*** | ***Cer^H215D^+24h*** |
| --- | --- | --- |
|  | 174 | 96 |
|  | 317 | 267 |
|  | 173 | 152 |
|  | 182 | 148 |
|  | 143 | 88 |
|  | 212 | 170 |
| **mean** | 200 | 153.5 |
| **SEM** | 25.04 | 26.32 |

Paired t-test

Figure 1G

| 1= | rolling |
| --- | --- |
| 2= | bending |
| 3= | stop and turn |
| 4= | stop |
| 5= | no response |
|  |  |

| reaction | 1 | 2 | 3 | 4 | 5 |
| --- | --- | --- | --- | --- | --- |
| 21-7-Gal4>UAS-Dicer2 | 0 | 0 | 4 | 19 | 37 |
| UAS-cerSdsRNA2 | 0 | 0 | 7 | 18 | 35 |
| 21-7-Gal4>UAS-Dicer2/UAS-cerSdsRNA2 | 0 | 0 | 26 | 15 | 19 |

| reaction | 1 | 2 | 3 | 4 | 5 |
| --- | --- | --- | --- | --- | --- |
| 21-7-Gal4>UAS-Dicer2 | 0 | 0 | 9 | 13 | 38 |
| UAS-cerSdsRNA1 | 0 | 0 | 17 | 14 | 34 |
| 21-7-Gal4>UAS-Dicer2/UAS-cerSdsRNA1 | 0 | 0 | 19 | 18 | 23 |

Statistical test used: Chi^2^-test

**FIGURE 2**

Figure 2A

length

|  | control | cerSP61 |
| --- | --- | --- |
|  | 13,049 | 8,441 |
|  | 10,729 | 9,701 |
|  | 14,549 | 9,6887 |
|  | 14,267 | 9,5303 |
|  | 12,067 | 8,4205 |
| **mean** | 12,93 | 9,156 |
| **SEM** | 0,7075 | 0,2977 |

Unpaired t-test

Figure 2B

|  | ***white^1118^*** | ***cerS^P61^*** |
| --- | --- | --- |
|  | 54791,2105 | 3913,9385 |
|  | 28216,5611 | 7048,62029 |
|  | 70757,3592 | 8252,89455 |
|  | 51032,1214 | 10830,7655 |
| **mean** | 51199 | 10831 |
| **SEM** | 8773 | 1435 |

Unpaired t-test

Figure 2D

Cer

|  | ***white^1118^*** | ***cerS^P61^*** |
| --- | --- | --- |
|  | 2,24936451 | 2,33768178 |
|  | 2,70047502 | 1,88071619 |
|  | 1,48522657 | 1,75903108 |
|  | 2,24293785 | 1,28347275 |
| **mean** | 2.17 | 1.815 |
| **SEM** | 0.252 | 0.2166 |

Unpaired t-test

Cer-PE

|  | ***white^1118^*** | ***cerS^P61^*** |
| --- | --- | --- |
|  | 2,3368606 | 1,81644285 |
|  | 3,39423557 | 1,92985819 |
|  | 2,13799433 | 1,52996083 |
|  | 3,1094995 | 1,18829708 |
| **mean** | 2.745 | 1.656 |
| **SEM** | 0.3013 | 0.1656 |

Unpaired t-test

Hex-Cer

|  | ***white^1118^*** | ***cerS^P61^*** |
| --- | --- | --- |
|  | 0,69618544 | 0,49007973 |
|  | 0,64169987 | 0,2067314 |
|  | 0,34518177 | 0,87383659 |
|  | 0,61070113 | 0,28205553 |
| **mean** | 0,5734 | 0,4632 |
| **SEM** | 0,07811 | 0,1494 |

Unpaired t-test

**Figure 3**

Length

|  | *cerS^WT^* | *FRT40A* | *lace^SK6^*, *FRT40A* | *cerS^H151D^* | *des1^KO^, FRT40A* |
| --- | --- | --- | --- | --- | --- |
|  | 17,576 | 20,764 | 9,266 | 5,5256 | 14,715 |
|  | 19,814 | 20,322 | 8,8817 | 5,2793 | 15,57 |
|  | 22,16 | 20,961 | 10,784 | 5,9755 | 12,008 |
|  | 18,752 | 18,387 | 7,679 | 4,3673 | 14,537 |
|  | 23,656 | 21,114 | 6,979 | 4,5506 | 15,627 |
|  | 22,732 |  | 9,873 | 4,5133 |  |
|  | 15,032 |  | 9,771 | 4,6941 |  |
| mean | 19,96 | 20,31 | 9,033 | 4,987 | 14,49 |
| SEM | 1,171 | 0,4987 | 0,4987 | 0,2308 | 0,6585 |

1-Way-ANOVA followed by Tukey´s multiple comparison test

|  | *FRT42D* | *cpeS^KO^, FRT42D* |
| --- | --- | --- |
|  | 13,779 | 11,692 |
|  | 18,828 | 9,649 |
|  | 18,988 | 11,466 |
|  | 17,024 | 9,347 |
|  | 16,276 | 10,506 |
|  | 16,011 | 10,257 |
|  | 17,475 | 10,44 |
| mean | 16,91 | 10,48 |
| SEM | 0,6786 | 0,3261 |

Unpaired t-test

Terminals

Length

|  | *cerS^WT^* | *FRT40A* | *lace^SK6^*, *FRT40A* | *cerS^H151D^* | *des1^KO^, FRT40A* |
| --- | --- | --- | --- | --- | --- |
|  | 17,576 | 20,764 | 9,266 | 5,5256 | 14,715 |
|  | 19,814 | 20,322 | 8,8817 | 5,2793 | 15,57 |
|  | 22,16 | 20,961 | 10,784 | 5,9755 | 12,008 |
|  | 18,752 | 18,387 | 7,679 | 4,3673 | 14,537 |
|  | 23,656 | 21,114 | 6,979 | 4,5506 | 15,627 |
|  | 22,732 |  | 9,873 | 4,5133 |  |
|  | 15,032 |  | 9,771 | 4,6941 |  |
| mean | 721,9 | 640,2 | 189,6 | 61,14 | 437,6 |
| SEM | 43,16 | 17,59 | 11,02 | 6,045 | 19,80 |

1-Way-ANOVA followed by Tukey´s multiple comparison test

|  | *FRT42D* | *cpeS^KO^, FRT42D* |
| --- | --- | --- |
|  | 252 | 232 |
|  | 474 | 153 |
|  | 484 | 221 |
|  | 366 | 141 |
|  | 400 | 203 |
|  | 337 | 180 |
|  | 419 | 212 |
| mean | 390,3 | 191,7 |
| SEM | 30,57 | 13,12 |

Unpaired t-test

**Figure 4**

Figure 4B

(dh)S

|  | ***white^1118^*** | ***cerS^P61^*** |
| --- | --- | --- |
|  | 15,650547 | 163,406419 |
|  | 18,4250946 | 139,850305 |
|  | 14,6523891 | 127,471629 |
|  | 18,5232468 | 123,344156 |
|  | 11,0014572 | 74,2341243 |
| **mean** | 15,65 | 125,7 |
| **SEM** | 1,389 | 14,63 |

Statistical test used: unpaired t-test

(dh)S1P

|  | ***white^1118^*** | ***cerS^P61^*** |
| --- | --- | --- |
|  | 0,065807 | 0,29237415 |
|  | 0,08132374 | 0,09641475 |
|  | 0,03433875 | 0,1411579 |
|  | 0,07586796 | 0,10503507 |
|  | 0,07169755 | 0,13952994 |
| **mean** | 0,06581 | 0,1549 |
| **SEM** | 0,008267 | 0,03552 |

Statistical test used: unpaired t-test

length

|  | *cerS^WT^* | *cerS^WT^; UAS-sk1* | *cerS^WT^; UAS-sk2* | *cerS^HD^* | *cerS^HD^*, *UAS-sk1* | *cerS^HD^*, *UAS-sk2* |
| --- | --- | --- | --- | --- | --- | --- |
|  | 17,576 | 12,072 | 11,537 | 5,5256 | 3,5018 | 1,6782 |
|  | 19,814 | 14,248 | 15,684 | 5,2793 | 3,4315 | 1,8114 |
|  | 22,16 | 15,84 | 17,996 | 5,9755 | 2,7002 | 1,456 |
|  | 18,752 | 15,667 | 13,398 | 4,3673 | 1,6121 | 1,1835 |
|  | 23,656 | 14,754 | 14,917 | 4,5506 | 3,3273 | 2,1271 |
|  | 22,732 | 15,81 | 14,651 | 4,5133 | 1,9698 | 0,9951 |
|  | 15,032 | 19,156 | 12,639 | 4,6941 |  | 1,2193 |
|  |  |  |  |  |  | 1,8881 |
| **mean** | 19,96 | 15,36 | 14,40 | 4,987 | 2,757 | 1,545 |
| **SEM** | 1,171 | 0,8070 | 0,8049 | 0,2308 | 0,3301 | 0,1397 |

1-Way-ANOVA followed by Dunnett´s multiple comparison.

termianls

|  | *cerS^WT^* | *cerS^WT^; UAS-sk1* | *cerS^WT^; UAS-sk2* | *cerS^HD^* | *cerS^HD^*, *UAS-sk1* | *cerS^HD^*, *UAS-sk2* |
| --- | --- | --- | --- | --- | --- | --- |
|  | 780 | 430 | 600 | 89 | 105 | 61 |
|  | 619 | 559 | 737 | 62 | 137 | 110 |
|  | 827 | 607 | 838 | 68 | 80 | 40 |
|  | 676 | 652 | 502 | 49 | 23 | 51 |
|  | 683 | 685 | 626 | 69 | 126 | 85 |
|  | 891 | 653 | 592 | 48 | 53 | 78 |
|  | 577 | 690 | 472 | 43 |  | 100 |
|  |  |  |  |  |  | 85 |
| **mean** | 721,9 | 610,9 | 623,9 | 61,14 | 87,33 | 76,25 |
| **SEM** | 43,16 | 34,72 | 48,34 | 6,045 | 17,93 | 8,497 |

1-Way-ANOVA followed by Dunnett´s multiple comparison.

Figure 4F

length

|  | *cerS^HD^* | *cerS^HD^*, *UAS-lace^dsRNA1^* | *cerS^HD^*, *UAS-sk2^dsRNA1^* | *cerS^HD^*, *UAS-tre1^dsRNA2^* | *cerS^HD^*, *UAS-sply* |
| --- | --- | --- | --- | --- | --- |
|  | 5,5256 | 5,8894 | 6,5229 | 3,1359 | 7,4035 |
|  | 5,2793 | 3,7376 | 2,1469 | 4,8178 | 5,2837 |
|  | 5,9755 | 3,889 | 4,4587 | 5,0594 | 6,4249 |
|  | 4,3673 | 5,1428 | 3,4642 | 5,9281 | 6,209 |
|  | 4,5506 | 4,7704 | 4,8576 | 3,5233 | 5,4521 |
|  | 4,5133 | 3,0048 | 3,9104 |  | 6,8977 |
|  | 4,6941 | 3,2396 | 5,3997 |  |  |
| **mean** | 4,987 | 4,239 | 4,394 | 4,493 | 6,278 |
| **SEM** | 0,2308 | 0,4000 | 0,5325 | 0,5132 | 0,3341 |

1-Way-ANOVA followed by Dunnett´s multiple comparison.

Terminals

|  | *cerS^HD^* | *cerS^HD^*, *UAS-lace^dsRNA1^* | *cerS^HD^*, *UAS-sk2^dsRNA1^* | *cerS^HD^*, *UAS-tre1^dsRNA2^* | *cerS^HD^*, *UAS-sply* |
| --- | --- | --- | --- | --- | --- |
|  | 89 | 78 | 86 | 24 | 160 |
|  | 62 | 49 | 27 | 101 | 96 |
|  | 68 | 39 | 36 | 83 | 94 |
|  | 49 | 65 | 32 | 62 | 108 |
|  | 69 | 64 | 71 | 30 | 61 |
|  | 48 | 37 | 56 |  | 158 |
|  | 43 | 27 | 151 |  |  |
| **mean** | 61,14 | 51,29 | 65,57 | 60,00 | 112,8 |
| **SEM** | 6,045 | 6,924 | 16,42 | 14,85 | 15,93 |

1-Way-ANOVA followed by Dunnett´s multiple comparison.

Figure 5

|  | C12-Cer |  | C16-Cer |  | C18-Cer |  | C20-Cer |  |
| --- | --- | --- | --- | --- | --- | --- | --- | --- |
|  | ***white^1118^*** | ***cerS^P61^*** | ***white^1118^*** | ***cerS^P61^*** | ***white^1118^*** | ***cerS^P61^*** | ***white^1118^*** | ***cerS^P61^*** |
|  | 0,0672985 | 0,74352922 | 0,0765561 | 0,80597456 | 0,15746067 | 0 | 0,9467338 | 0,44119521 |
|  | 0,10893352 | 0,38564648 | 0,21655731 | 0,47847148 | 0,17535394 | 0 | 1,1289901 | 0,51165766 |
|  | 0,04874872 | 0,46167379 | 0,11159538 | 0,3666941 | 0,13033855 | 0 | 0,63693144 | 0,49758957 |
|  | 0,05984833 | 0,29908123 | 0,18506107 | 0,29648593 | 0,18230462 | 0 | 0,97147848 | 0,38469959 |
| mean | 0,07121 | 0,4725 | 0,1474 | 0,4869 | 0,1614 | 0,000 | 0,9210 | 0,4588 |
| SEM | 0,01314 | 0,09626 | 0,03228 | 0,1128 | 0,01159 | 0,000 | 0,1029 | 0,02901 |

|  | C22-Cer |  | C24-Cer |  |
| --- | --- | --- | --- | --- |
|  | ***white^1118^*** | ***cerS^P61^*** | ***white^1118^*** | ***cerS^P61^*** |
|  | 0,93574708 | 0,34698279 | 0,06556835 | 0 |
|  | 1,00393309 | 0,50494057 | 0,06670706 | 0 |
|  | 0,52255514 | 0,43307362 | 0,03505735 | 0 |
|  | 0,79825469 | 0,30320601 | 0,04599065 | 0 |
| mean | 0,8151 | 0,3971 | 0,05333 | 0,000 |
| SEM | 0,1065 | 0,04496 | 0,007727 | 0,000 |

Statistical test used: unpaired t-test

Figure 5D

length

|  | *cerS^WT^* | *cerS^H151D^* | *cerS^H151D^+ UAS-Cers5* | *cerS^H151D^+ UAS-Cers1* | *cerS^H151D^+ UAS-Cers4* | *cerS^H151D^+ UAS-Cers2* |
| --- | --- | --- | --- | --- | --- | --- |
|  | 17,576 | 5,5256 | 6,98 | 16,238 | 19,888 | 19,235 |
|  | 19,814 | 5,2793 | 7,158 | 14,242 | 25,057 | 19,227 |
|  | 22,16 | 5,9755 | 5,834 | 14,322 | 17,152 | 19,767 |
|  | 18,752 | 4,3673 | 9,094 | 13,41 | 18,6 | 19,853 |
|  | 23,656 | 4,5506 | 10,67 | 15,531 | 19,697 | 17,455 |
|  | 22,732 | 4,5133 | 9,046 | 17,875 | 22,321 | 19,779 |
|  | 15,032 | 4,6941 | 9,689 | 12,879 | 21,954 | 19,167 |
|  |  |  |  |  |  | 18,516 |
| mean | 19,96 | 4,987 | 8,353 | 14,93 | 20,67 | 19,12 |
| SEM | 1,171 | 0,2308 | 0,6518 | 0,6566 | 0,9981 | 0,2852 |

1-Way-ANOVA followed by Dunnett´s multiple comparison.

Terminals

|  | *cerS^WT^* | *cerS^H151D^* | *cerS^H151D^+ UAS-Cers5* | *cerS^H151D^+ UAS-Cers1* | *cerS^H151D^+ UAS-Cers4* | *cerS^H151D^+ UAS-Cers2* |
| --- | --- | --- | --- | --- | --- | --- |
|  | 780 | 89 | 116 | 499 | 742 | 664 |
|  | 619 | 62 | 139 | 511 | 852 | 623 |
|  | 827 | 68 | 121 | 363 | 655 | 487 |
|  | 676 | 49 | 158 | 492 | 695 | 530 |
|  | 683 | 69 | 220 | 421 | 736 | 515 |
|  | 891 | 48 | 169 | 402 | 901 | 769 |
|  | 577 | 43 | 190 | 337 | 823 | 621 |
|  |  |  |  |  |  | 513 |
| mean | 721,9 | 61,14 | 159,0 | 432,1 | 772,0 | 590,3 |
| SEM | 43,16 | 6,045 | 14,21 | 26,34 | 33,61 | 34,16 |

1-Way-ANOVA followed by Dunnett´s multiple comparison.

**Figure 6**

Figure 6A

% of total ceramide

|  | *white^1118^* | tub > mCerS5 |
| --- | --- | --- |
|  | 3,40345443 | 15,7920483 |
|  | 8,0192303 | 20,1599932 |
|  | 7,51369388 | 15,4549027 |
|  | 8,25083371 | 14,62197 |
| mean | 6,797 | 16,51 |
| SEM | 1,142 | 1,242 |

Statistical test used in A: Mann-Whitney test

% of total cer-PE

|  | *white^1118^* | tub > mCerS5 |
| --- | --- | --- |
|  | 0 | 29,4533915 |
|  | 0 | 29,1751766 |
|  | 0 | 28,8071967 |
|  | 0,20580041 | 25,6169191 |
| mean | 0,05145 | 28,26 |
| SEM | 0,05145 | 0,8920 |

Statistical test used in A: Mann-Whitney test

Pie diagrams white

| white cer |  | white cer-PE |  |
| --- | --- | --- | --- |
| C12-Cer | 0,07120727 | C16-Cer-PE | 0,00159984 |
| C16-Cer | 0,14744247 | C18-Cer-PE | 0,26302659 |
| C18-Cer | 0,16136445 | C20-CerPE | 1,23989227 |
| C20-Cer | 0,92103346 | C22-CerPE | 1,081122 |
| C22-Cer | 0,8151225 | C24-CerPE | 0,15900681 |
| C24-Cer | 0,05333085 |  |  |

Pie diagrams tub>UAS-mCerS5

| white cer |  | white cer |  |
| --- | --- | --- | --- |
| C12-Cer | 0,0541993 | C16-Cer-PE | 0,52017529 |
| C16-Cer | 0,15405397 | C18-Cer-PE | 0,13685515 |
| C18-Cer | 0,19904923 | C20-CerPE | 0,61616903 |
| C20-Cer | 0,40747393 | C22-CerPE | 0,48917314 |
| C22-Cer | 0,24418179 | C24-CerPE | 0,15900681 |
| C24-Cer | 0,00800929 |  |  |

Figure 6C

length

|  | *control* | *UAS-mCers5* | *UAS-mCers1* | *UAS-mCers4* | *UAS-mCers2* |
| --- | --- | --- | --- | --- | --- |
|  | 22,597 | 19,816 | 22,093 | 22,37 | 17,905 |
|  | 23,077 | 10,296 | 23,393 | 21,474 | 22,941 |
|  | 24,214 | 15,906 | 21,476 | 20,495 | 17,25 |
|  | 20,327 | 14,195 | 20,95 | 22,559 | 19,123 |
|  | 20,101 | 15,488 | 24,475 | 21,08 | 24,977 |
| mean | 22,06 | 15,14 | 22,48 | 21,60 | 20,44 |
| SEM | 0,8001 | 1,532 | 0,6447 | 0,3886 | 1,503 |

1-Way-ANOVA followed by Dunnett´s multiple comparison

terminals

|  | *control* | *UAS-mCers5* | *UAS-mCers1* | *UAS-mCers2* | *UAS-mCers4* |
| --- | --- | --- | --- | --- | --- |
|  | 838 | 523 | 666 | 556 | 632 |
|  | 900 | 309 | 736 | 645 | 699 |
|  | 947 | 474 | 628 | 548 | 599 |
|  | 746 | 392 | 713 | 627 | 586 |
|  | 772 | 433 | 820 | 820 | 684 |
| mean | 840,6 | 426,2 | 712,6 | 639,2 | 640,0 |
| SEM | 37,76 | 36,47 | 32,72 | 49,05 | 22,45 |

1-Way-ANOVA followed by Dunnett´s multiple comparison

**Figure S1**

Figure S1B

Length

|  | *CerS^WT^* | *cerS^KO^* | *rescue* |
| --- | --- | --- | --- |
|  | 2,2588 | 1,2689 | 1,584 |
|  | 2,7425 | 1,6226 | 2,0997 |
|  | 2,348 | 1,4271 | 2,1655 |
|  | 2,0436 | 1,6953 | 2,5646 |
|  | 2,2916 | 1,3973 | 2,2253 |
|  | 1,9468 | 1,2139 | 2,0729 |
|  | 2,6335 | 1,6898 | 1,3553 |
|  | 3,1832 | 1,5467 | 2,1111 |
|  | 2,865 | 1,1674 | 2,2275 |
|  | 2,211 | 1,4324 | 2,1588 |
|  | 2,3641 |  | 1,3705 |
|  |  |  | 2,2241 |
|  |  |  | 2,1826 |
| **mean** | 2,444 | 1,446 | 2,026 |
| **SEM** | 0,1122 | 0,06043 | 0,1000 |

Kruskal-Wallis test followed by a Dunn´s multiple comparison test

terminals

|  | *CerS^WT^* | *cerS^KO^* | *rescue* |
| --- | --- | --- | --- |
|  | 26 | 15 | 27 |
|  | 26 | 16 | 28 |
|  | 27 | 21 | 35 |
|  | 20 | 21 | 35 |
|  | 35 | 14 | 30 |
|  | 25 | 17 | 30 |
|  | 42 | 16 | 27 |
|  | 32 | 20 | 27 |
|  | 30 | 14 | 33 |
|  | 26 | 16 | 30 |
|  | 40 |  | 22 |
|  |  |  | 26 |
|  |  |  | 30 |
| **mean** | 29,91 | 17,00 | 29,21 |
| **SEM** | 2,034 | 0,8563 | 0,9445 |

1-Way-ANOVA followed by Tukey´s multiple comparison test

Figure S1C

Length

|  | *CerS^WT^* | *cerS^KO^* | *cerS^KO^+ UAS-cerS* |
| --- | --- | --- | --- |
|  | 3,3361 | 2,7396 | 4,1345 |
|  | 3,8322 | 2,5745 | 4,5388 |
|  | 5,1148 | 2,8424 | 3,6619 |
|  | 4,4621 | 2,5282 | 3,9131 |
|  | 5,2343 | 2,5929 | 3,876 |
| **mean** | 4,396 | 2,656 | 4,025 |
| **SEM** | 0,3650 | 0,05860 | 0,1488 |

1-Way-ANOVA followed by Tukey´s multiple comparison test

Terminals

|  | *CerS^WT^* | *cerS^KO^* | *cerS^KO^+ UAS-cerS* |
| --- | --- | --- | --- |
|  | 185 | 60 | 242 |
|  | 300 | 49 | 242 |
|  | 491 | 65 | 174 |
|  | 363 | 53 | 300 |
|  | 443 | 38 | 255 |
| **mean** | 356,4 | 53,00 | 242,6 |
| **SEM** | 53,95 | 4,658 | 20,19 |

1-Way-ANOVA followed by Tukey´s multiple comparison test

Figure S1D

Length

|  | cerSWT |  |  | cerSKO |  |  |
| --- | --- | --- | --- | --- | --- | --- |
|  | LII | LIIIf | LIIIw | LII | LIIIf | LIIIw |
|  | 8,063 | 13,864 | 17,576 | 5,7805 | 5,8292 | 3,9382 |
|  | 10,296 | 13,061 | 19,814 | 4,7715 | 7,2964 | 5,4918 |
|  | 9,725 | 11,378 | 22,16 | 4,3698 | 8,192 | 4,7183 |
|  | 10,905 | 9,716 | 18,752 | 5,5459 | 7,2589 | 5,5593 |
|  | 8,814 | 13,003 | 23,656 | 5,5362 | 8,37 | 5,6464 |
|  | 8,045 |  | 22,732 | 5,6382 |  | 5,7764 |
|  | 7,1 |  | 15,032 | 4,5729 |  | 5,3473 |
| mean | 8,993 | 12,20 | 19,96 | 5,174 | 7,389 | 5,211 |
| SEM | 0,5180 | 0,7416 | 1,171 | 0,2195 | 0,4509 | 0,2484 |

2-Way-ANOVA followed by Sidak´s multiple comparison test

Terminals

|  | cerSWT |  |  | cerSKO |  |  |
| --- | --- | --- | --- | --- | --- | --- |
|  | LII | LIIIf | LIIIw | LII | LIIIf | LIIIw |
|  | 459 | 655 | 780 | 184 | 147 | 48 |
|  | 553 | 602 | 619 | 157 | 150 | 69 |
|  | 502 | 550 | 827 | 165 | 211 | 48 |
|  | 540 | 510 | 676 | 193 | 177 | 91 |
|  | 436 | 686 | 683 | 163 | 198 | 104 |
|  | 405 |  | 891 | 189 |  | 127 |
|  | 378 |  | 577 | 141 |  | 80 |
| mean | 467,6 | 600,6 | 721,9 | 170,3 | 176,6 | 81,00 |
| SEM | 25,23 | 32,42 | 43,16 | 7,187 | 12,70 | 10,98 |

2-Way-ANOVA followed by Sidak´s multiple comparison test

Figure S1E

| Statistical test used: One-Way ANOVA with Tukey´s comparison test  Figure 1E  length   \|  \| ***CerS^KO^*** \| ***CerS^KO^+24h*** \| \| --- \| --- \| --- \| \|  \| 8,4134 \| 8,3707 \| \|  \| 8,0708 \| 8,192 \| \|  \| 7,2589 \| 6,4732 \| \|  \| 7,2964 \| 6,5024 \| \|  \| 6,1314 \| 5,8292 \| \| **mean** \| 7.434 \| 7.074 \| \| **SEM** \| 0.3944 \| 0.5084 \| |  |  |  |
| --- | --- | --- | --- | --- | --- | --- | --- | --- | --- | --- | --- | --- | --- | --- | --- | --- | --- | --- | --- | --- | --- | --- | --- | --- | --- | --- | --- |

Paired t-test

Terminals

|  | ***CerS^KO^*** | ***CerS^KO^+24h*** |
| --- | --- | --- |
|  | 198 | 171 |
|  | 211 | 176 |
|  | 177 | 116 |
|  | 150 | 106 |
|  | 147 | 143 |
| **mean** | 176.6 | 142.2 |
| **SEM** | 12.7 | 14.09 |

Paired t-test

**Figure S2**

Figure S2B

length

|  | *cerS^KO^* | *cerS^KO^;UAS-p35* | *cerS^KO^;;UAS-p35* |
| --- | --- | --- | --- |
|  | 3,9382 | 3,3366 | 4,5236 |
|  | 5,4918 | 3,6863 | 4,2336 |
|  | 4,7183 | 2,8254 | 3,8649 |
|  | 5,5593 | 4,5557 | 4,1321 |
|  | 5,6464 | 4,4802 | 5,0841 |
|  | 5,7764 | 4,8843 | 6,2381 |
|  | 5,3473 | 3,868 | 4,1058 |
| mean | 5,211 | 3,948 | 4,597 |
| SEM | 0,2484 | 0,2777 | 0,3110 |

1-Way-ANOVA followed by Dunnett´s multiple comparison test.

terminals

|  | *cerS^KO^* | *cerS^KO^;UAS-p35* | *cerS^KO^;;UAS-p35* |
| --- | --- | --- | --- |
|  | 48 | 31 | 66 |
|  | 69 | 32 | 47 |
|  | 48 | 21 | 60 |
|  | 91 | 43 | 75 |
|  | 104 | 49 | 64 |
|  | 127 | 42 | 63 |
|  | 80 | 27 | 34 |
| mean | 81,00 | 35,00 | 58,43 |
| SEM | 10,98 | 3,761 | 5,149 |

1-Way-ANOVA followed by Dunnett´s multiple comparison test.

**Figure S3**

Length

|  | *control^dsRNA^* | *UAS-spt1^dsRNA1^* | *UAS-spt1^dsRNA2^* | *UAS-lace^dsRNA1^* | *UAS-lace^dsRNA2^* | *UAS-αKR^dsRNA1^* | *UAS-αKR^dsRNA2^* | *UAS-cerS^dsRNA1^* | *UAS-cerS^dsRNA2^* | *UAS-des1^dsRNA^* | *UAS-cpes^dsRNA1^* | *UAS-cpes^dsRNA2^* | *UAS-glcT^dsRNA1^* | *UAS-glcT^dsRNA2^* |
| --- | --- | --- | --- | --- | --- | --- | --- | --- | --- | --- | --- | --- | --- | --- |
|  | 20,045 | 14,018 | 15,007 | 11,64 | 14,161 | 20,881 | 14,596 | 10,366 | 10,507 | 14,845 | 12,664 | 15,416 | 19,057 | 20,285 |
|  | 20,919 | 14,361 | 14,273 | 12,67 | 13,45 | 18,948 | 16,93 | 9,299 | 9,751 | 19,095 | 17,82 | 15,134 | 17,6 | 17,637 |
|  | 20,8 | 13,292 | 11,111 | 11,852 | 14,523 | 18,686 | 15,777 | 9,965 | 11,677 | 16,58 | 19,842 | 15,36 | 18,6 | 18,867 |
|  | 23,078 | 14,412 | 10,801 | 14,839 | 12,088 | 16,939 | 17,381 | 11,357 | 10,202 | 16,574 | 15,5655 | 15,271 | 21,247 | 18,151 |
|  | 22,708 | 13,617 | 11,801 | 12,096 | 14,638 | 17,516 | 18,558 | 11,588 | 10,69 | 17,155 | 14,651 | 15,149 | 15,518 | 19,483 |
|  | 23,01 | 12,315 | 11,391 | 14,249 | 13,353 | 17,157 | 22,356 | 8,835 | 10,478 | 17,777 | 15,14 | 13,823 | 19,045 | 22,231 |
|  | 21,549 | 13,482 | 13,949 | 15,422 | 12,104 | 16,554 | 21,595 | 9,831 | 9,597 | 15,632 | 13,231 | 11,1791 |  | 21,67 |
| mean | 21,73 | 13,64 | 12,62 | 13,25 | 13,47 | 18,10 | 18,17 | 10,18 | 10,41 | 16,81 | 15,56 | 14,48 | 18,51 | 19,76 |
| SEM | 0,4579 | 0,2743 | 0,6540 | 0,5867 | 0,4006 | 0,5725 | 1,092 | 0,3828 | 0,2599 | 0,5263 | 0,9544 | 0,5871 | 0,7717 | 0,6546 |

Kruskal-Wallis with Dunn´s multiple comparison test.

Terminals

|  | *control^dsRNA^* | *UAS-spt1^dsRNA1^* | *UAS-spt1^dsRNA2^* | *UAS-lace^dsRNA1^* | *UAS-lace^dsRNA2^* | *UAS-αKR^dsRNA1^* | *UAS-αKR^dsRNA2^* | *UAS-cerS^dsRNA1^* | *UAS-cerS^dsRNA2^* | *UAS-des1^dsRNA^* | *UAS-cpes^dsRNA1^* | *UAS-cpes^dsRNA2^* | *UAS-glcT^dsRNA1^* | *UAS-glcT^dsRNA2^* |
| --- | --- | --- | --- | --- | --- | --- | --- | --- | --- | --- | --- | --- | --- | --- |
|  | 677 | 413 | 555 | 426 | 347 | 768 | 474 | 271 | 252 | 549 | 425 | 457 | 631 | 557 |
|  | 687 | 432 | 501 | 436 | 460 | 641 | 637 | 293 | 254 | 685 | 628 | 504 | 513 | 424 |
|  | 687 | 358 | 380 | 406 | 401 | 752 | 646 | 230 | 354 | 602 | 767 | 551 | 573 | 561 |
|  | 764 | 598 | 345 | 349 | 501 | 575 | 633 | 343 | 253 | 577 | 557 | 422 | 620 | 442 |
|  | 780 | 409 | 431 | 430 | 376 | 666 | 576 | 373 | 311 | 623 | 536 | 430 | 367 | 484 |
|  | 829 | 348 | 420 | 397 | 456 | 580 | 723 | 243 | 243 | 675 | 582 | 371 | 627 | 627 |
|  | 766 | 415 | 556 | 421 | 628 | 521 | 704 | 266 | 233 | 512 | 461 | 317 |  | 593 |
| mean | 741,4 | 424,7 | 455,4 | 409,3 | 452,7 | 643,3 | 627,6 | 288,4 | 271,4 | 603,3 | 565,1 | 436,0 | 555,2 | 526,9 |
| SEM | 22,01 | 31,20 | 31,56 | 11,29 | 35,47 | 35,02 | 31,49 | 19,78 | 16,68 | 23,99 | 42,65 | 29,64 | 41,88 | 29,32 |

Kruskal-Wallis with Dunn´s multiple comparison test.

**Figure S4**

Length

|  | control | *ppk-Gal4 > cerS^dsRNA1^* | *ppk-Gal4 > cerS^dsRNA2^* | *A58-Gal4 > UAS-cerS^dsRNA1^* | *A58-Gal4 > UAS-cerS^dsRNA2^* | *repo-Gal4 > UAS-cerS^dsRNA1^* |
| --- | --- | --- | --- | --- | --- | --- |
|  | 20,045 | 10,366 | 10,507 | 20,434 | 26,281 | 22,619 |
|  | 20,919 | 9,299 | 9,751 | 18,657 | 21,117 | 22,567 |
|  | 20,8 | 9,965 | 11,677 | 20,99 | 20,782 | 25,38 |
|  | 23,078 | 11,357 | 10,202 | 20,518 | 24,471 | 23,564 |
|  | 22,708 | 11,588 | 10,69 | 20,808 | 20,855 | 19,993 |
|  | 23,01 | 8,835 | 10,478 |  |  |  |
|  | 21,549 | 9,831 | 9,597 |  |  |  |
| mean | 21,73 | 10,18 | 10,41 | 20,28 | 22,70 | 22,82 |
| SEM | 0,4579 | 0,3828 | 0,2599 | 0,4182 | 1,130 | 0,8719 |

1-Way-ANOVA followed by Dunnett´s multiple comparison.

Terminals

|  | control | *ppk-Gal4 > cerS^dsRNA1^* | *ppk-Gal4 > cerS^dsRNA2^* | *A58-Gal4 > UAS-cerS^dsRNA1^* | *A58-Gal4 > UAS-cerS^dsRNA2^* | *repo-Gal4 > UAS-cerS^dsRNA1^* |
| --- | --- | --- | --- | --- | --- | --- |
|  | 20,045 | 677 | 271 | 252 | 940 | 612 |
|  | 20,919 | 687 | 293 | 254 | 626 | 636 |
|  | 20,8 | 687 | 230 | 354 | 655 | 760 |
|  | 23,078 | 764 | 343 | 253 | 760 | 716 |
|  | 22,708 | 780 | 373 | 311 | 712 | 730 |
|  | 23,01 | 829 | 243 | 243 |  |  |
|  | 21,549 | 766 | 266 | 233 |  |  |
| mean | 21,73 | 741,4 | 288,4 | 271,4 | 738,6 | 690,8 |
| SEM | 22,01 | 19,78 | 16,68 | 55,41 | 28,44 | 44,83 |

1-Way-ANOVA followed by Dunnett´s multiple comparison.

**Figure S5**

Length

|  | *CerS^WT^* | *cerS^H215D^* | *cerS^H215D^+ C16:ceramide* | *cerS^H215D^+ C18:ceramide* | *cerS^HD^+ C22-Cer* |
| --- | --- | --- | --- | --- | --- |
|  | 17,576 | 5,5256 | 5,5519 | 7,0771 | 7,817 |
|  | 19,814 | 5,2793 | 4,2277 | 6,8112 | 7,8977 |
|  | 22,16 | 5,9755 | 5,1111 | 6,8267 | 6,2035 |
|  | 18,752 | 4,3673 | 3,3721 | 6,7566 | 5,8235 |
|  | 23,656 | 4,5506 | 3,4818 | 4,6584 | 6,364 |
|  | 22,732 | 4,5133 | 3,6911 | 5,1741 | 7,6364 |
|  | 15,032 | 4,6941 | 5,6026 | 6,5461 | 7,3349 |
| **mean** | **19.96** | **4,987** | **4,434** | **6,264** | **7,011** |
| **SEM** | **1.171** | **0,2308** | **0,3685** | **0,3574** | **0,3241** |

Kruskal-Wallis test followed by a Dunn´s multiple comparison test

Terminals

|  | *CerS^WT^* | | *cerS^H215D^* | *cerS^H215D^+ C16:ceramide* | *cerS^H215D^+ C18:ceramide* | *cerS^HD^+ C22-Cer* |
| --- | --- | --- | --- | --- | --- | --- |
|  | | 780 | 89 | 109 | 128 | 225 |
|  | | 619 | 62 | 51 | 111 | 196 |
|  | | 827 | 68 | 74 | 108 | 126 |
|  | | 676 | 49 | 35 | 177 | 146 |
|  | | 683 | 69 | 46 | 31 | 82 |
|  | | 891 | 48 | 48 | 59 | 176 |
|  | | 577 | 43 | 55 | 124 | 143 |
| mean | | **721.9** | 61,14 | 59,71 | 105,4 | 156,3 |
| SEM | | **43.16** | 6,045 | 9,345 | 18,07 | 17,87 |

1-Way-ANOVA followed by Dunnett´s multiple comparison.
