# Supplementary material for: Individual lipid alterations at the origin of neuronal Ceramide Synthase defects
Source: PLoS Genet. 2025 Sep 25;21(9):e1011880. doi: 10.1371/journal.pgen.1011880 (PMC12500085; doi:10.1371/journal.pgen.1011880)
Supplement: Attachment — Submitted filename: Comments to Authors.docx [file pgen.1011880.s008.docx]

Mutations in ceramide synthase 1 gene leads to rare genetic disorder progressive myoclonic epilepsy type 8 (PME8) in humans. Patients with PME8 show progressive neurological deterioration, development of seizures and cognitive impairment. However, it remains unclear how loss of CerS function promotes neurodegeneration, and what lipids are directly involved in this process. In this study Ziegler et al., 2024 have found that expression of disease-causing *cerS* allele in Drosophila sensory neurons resulted in early onset of dendrite loss. They also found reduction of dendrite complexity in several other de novo sphingolipid biosynthetic mutant or knockdown backgrounds, including *spt, lace, des1, and cpes*. Lipidomic analysis cerS^p61^ mutants revealed significant reduction of ceramide phosphoethanolamine (Cer-PE), and significant accumulation of long chain bases dihydrosphingosine (dh)S and dihydrosphingosine-1P (dh)S1P. Authors show that sphingosine kinase overexpression in cerS^H215D^ background further reduce the neurite complexity indicating toxic effects of S1P in neurons. Reduction of S1P levels by overexpression of S1P lyase mildly improve the neurite complexity. Lipidomic analysis further revealed selective accumulation of C12 and C16 ceramides and reduction of C18-24 ceramides in cerS mutants. Genetic rescue experiments showed that mouse ceramide synthases that specifically synthesize C18-C20 ceramides (mCerS2 and mCerS4) but not C16 ceramides (mCerS5) rescued the CerS mutant c4da dendrite morphology. Further feeding larvae with C18 ceramides but not C16 ceramides significantly marginally improves dendrite terminals. Taken together, this study provides important details about altered sphingolipid metabolites upon loss of cerS and their effects on neurodegeneration.

Overall, this a nice study about neuronal dendritic arborization and the impact of ceramide synthase gene function in this process. The experiments are well executed, and interpretations are largely consistent with results. However, there are several points that need to be addressed before the article is published.

There is a conundrum arising out of the argument put forth by the authors:

They claim that conversion of accumulated dihydrosphingosine to dihydrosphingosine 1-phosphate in the CerS^H215D^ mutant background is a major contributor to dendritic pathology observed in the mutant. When they express mCerS5 they see minimal rescue of the phenotype. If the mCerS5 is active in the transgenic animal, it should consume the accumulated LCBs in CerS mutant background, and the reduced LCBs should afford good rescue if accumulated LCB and LCBP is indeed responsible for dendritic length and branching phenotype. The authors have to confirm that mCerS5 is indeed active in the transgenic animals. If the enzyme is indeed active, it does not answer the conundrum but at least the authors would have an answer to a caveat that is raised by their results. Since it is difficult to work with MARCM clone generated tissues, they should ensure that expression of mCERS1 in a wild-type background is able to increase C16 ceramide which would mean mCERS1 expression in the *cerS* mutant background is indeed capable of decreasing the LCB and LCBPs. If the argument is Cer-PE is the major determinant of dendritic arborization function it is surprising that an SPT null MARCM clone that should have the most severe reduction in Cer-PE is not as defective as *cerS* mutant.

In the study the authors have argued that their *Drosophila* CerS^H215D^ mutant is a model for the study of PME8 a disease caused by mutations in *CerS 1* gene. That gene should have been their first choice from mammalian genes to rescue and study the sphingolipid profile. Yet, while they have used three of the other mouse genes for rescue, they have neither used *CerS 1* for rescue experiments nor discussed the rationale for not using it in the study.

Reduction of CerPE levels affects dendrite morphology. However, it will be interesting to see whether reduced levels of hexosylceramides/glucosylceramides also affect the dendrite morphology. Glucosylceramide synthase (GlcT) knockdown may answer this question.

Recently Drosophila G protein coupled receptor Tre1 was shown to be a potential S1P receptor (Jiakun Chen et al 2024), the authors could try to knockdown this receptor and see if c4da dendrite phenotype restores in cerS mutants.

Do you see any reduction in mature c4da neuron count in cerS mutants compared to controls? What are the other cells in that are brightly labelled in Supp Fig.2E (left/right corners).

There are several issues that can be addressed by editing the manuscript appropriately.

Lines 56-57: seems like an incorrect statement The refences cited do not provide data or support the notion that myoclonic seizures appear to be related to loss of CerS activity in glia. It should be noted that CerS1 has been shown to be expressed both in neurons and glia in mammals and the mutations have effects in the whole animals include neurons, glia and the liver.

Lines 63-64: The reviewers could not find any information indicating that epilepsy and the complex phenotype associated with the heterozygous loss of chromosome 1q21 is classified as PME8. Please provide the proper reference or state this in a separate sentence as a related CerS related disorder.

Lines 104-105: Cer^H215D^ is not a synonymous mutation to Human Myoclonus Epilepsy 8 patient mutation. The original report in EMBO in 2009 by one of the co-authors generated this allele as an active site mutant. The generation and use of Cer^H215D^ as a PME8 mutation (as the authors claim in the text) is puzzling. The authors have taken the trouble of generating the mutation and using in vivo recombination to generate an allele that is driven from the genomic locus. If the intention of the authors was to study HME8 mutation and its pathogenesis they should have generated Cer^H215Q^ allele instead of CerH215D. While the enzymatic activity is compromised in these patients it is not known if they are true null alleles, whereas the Cer^H215D^ has been shown to behave like null in this and previous study. Therefore, the authors are advised to remove this statement since their mutant does not reproduce the exact molecular replacement found in PME8 patients. Similarly, lines 118 and 119 needs to be modified.

Also, in figure 1A hCerS1^H183C^ should be corrected to hCerS1^H183Q^.

Lines 315-316: HSAN1 neuropathy is a dominant condition, which means phenotypes are seen in heterozygous patients. PME8 is caused by synonymous or non-synonymous homozygous mutations in CERS1. Homozygous mutations of SPT are lethal in mice and other organisms. Also, it is well established that the mutant forms of the enzymes demonstrate altered substrate specificity and produce deoxysphingolipids from the altered substrate utilization (alanine and glycine) that could be toxic. It is advised the authors edit lines 306-316.

Line 235: Please edit ‘out’ to our

Line 313: Please edit ‘spingoid to sphingoid

Line 322: Please edit ‘trough’ to through
