# Supplementary material for: Individual lipid alterations at the origin of neuronal Ceramide Synthase defects
Source: PLoS Genet. 2025 Sep 25;21(9):e1011880. doi: 10.1371/journal.pgen.1011880 (PMC12500085; doi:10.1371/journal.pgen.1011880)
Supplement: Attachment — Submitted filename: Comments_to_Authors_FINAL.docx [file pgen.1011880.s010.docx]

We thank the reviewers for their positive and valuable comments on our manuscript. During the revision we performed extensive experimentation and added the following experiments to our manuscript, resulting in 12 new figures or figure panels:

- Phenotype of c4da neurons in heterozygous *cerS^KO^* mutants (new Fig. S1A)
- Analysis of cell maintenance upon *cerS* KD (new Fig. S2D-H)
- Analysis of dendrite morphology in the *cerS^P61^* mutant (new Fig. 2B)
- Impact of *glcT* KD (new panel in Fig. S3)
- Quantification of HexCer (new panel in Fig. 2D)
- Impact of *cerS* KD in glia or epidermis (new Fig. S4)
- Analysis of *UAS-SK1* or *UAS-SK2* overexpression in *cerS^WT^* MARCM clones revealing the dose-dependent effect of (dh)S1P/S1P on c4da dendritic morphology (new panels in Fig. 4C/D)
- Analysis of the following double mutants *cerS^H215D^, UAS-lace^dsRNA^* or *cerS^H215D^, UAS-cepS^dsRNA^* or *cerS^H215D^, UAS-tre1^dsRNA^* (new panels in Fig. 4E/F)
- Quantitative analysis of dendrite morphology in *UAS-mCerS1*expressing *cerS^H215D^* mutant neurons (new panels in Fig. 5 C/D)
- Analysis of the ceramide species composition in *UAS-CerS5* overexpressing animals (new Fig. 6A)
- We overexpressed *UAS-mCerS5*, *UAS-mCerS1*, *UAS-CerS4*, or *UAS-mCerS2* to study the effect of a shift in ceramide species composition (new Fig. 6 B/D)
- We analyzed how feeding of C22-Cer would act on *cerS^H215D^* mutant MARCM clones (new panels in Fig. S6A/B)

In addition, we have modified our manuscript to include the new experimental data and to implement the reviewers' suggestions. We trust that the reviewers will be satisfied by this extensive revision and will support the publication of this strengthened manuscript.

Our point-by-point responses to all the reviewers' comments can be found below. In red, we highlighted new passages in the manuscript.

Reviewer #1: PLOS genetics comments:

The Reviewer recognizes that our findings enhance our understanding of the mechanism underlying sphingolipid dysregulation associated with PME8”. She/ he also acknowledges our detailed analyses of the changes in sphingolipid metabolites in CerS mutants […]”. We thank the Reviewer for her/ his positive comments. In addition, Reviewer 1 indicates that we should clarify better the relationship between the changes in CerPE, C18-C24 ceramide, and (dh)S1P amounts (for instance by genetic double mutant analyses and lipidomics analysis). We thank the reviewer for this suggestion and have now included this analysis. Please, find below the response to this point and to the other comments of Reviewer 1.

Comments:
1. In Fig1, does CerS activity only affect dendritic morphology of da neurons? How about other types of neurons? In other words, is “CerS mutation causes dendritic defect” a general or da-neuron-specific issue?

In fact, in Drosophila *cerS* has already been identified in screens as a gene that influences the morphology of nerve cells in the central nervous system. In the introduction, we mentioned the vLNs neurons, whose dendrite morphology also dependents on CerS expression. However, we expanded this to (p.4, l. 116-120) ” Furthermore, knockdown of *cerS* in *Drosophila* ventral lateral neurons (LNv´s), located in the larval CNS and in neurons forming the Mushroom bodies of the adult CNS, reduces the amount of neurites and leads to targeting defects [(25,26)](https://sciwheel.com/work/citation?ids=6829336,4823145&pre=&pre=&suf=&suf=&sa=0,0&dbf=0&dbf=0) suggesting a conserved role of CerS in the establishment or maintenance of neuronal morphology.”

A reduction in the dendrites of Purkinje cells was also observed in cerS1-deficient mice. We mentioned this initially in the introduction but expanded this part to also highlight the role of CerS in CNS neurons (p.4, l.106-109): “In these mutants, dendrite degeneration of the cerebellar Purkinje cells in the central nervous system (CNS) was observed and a causal link between general LCBs accumulation and dendritic degeneration established”

2. This leads to the next comment: For general audiences, it will be helpful to explain in the introduction why the dendritic morphology of the c4da neuron is a suitable model to study PEM8.

We agree with the reviewer that a detailed explanation of why c4da are a valuable model to study the metabolic origin of PME8 and have added the following paragraph: (p4/5 l. 123-133): “Here, we used *Drosophila* class four dendritic arborization (c4da) neurons to study the effects of CerS dysfunction on neurons. The complex dendrites of the c4da neurons are housed directly under the transparent cuticle of the larva which facilitates *in vivo* imaging [(27)](https://sciwheel.com/work/citation?ids=12023346&pre=&suf=&sa=0&dbf=0). They can be visualized and genetically manipulated with highly specific Gal4 driver lines, allowing a detailed quantitative analysis of their dendritic morphology in immobilized, fully intact and living animals. Due to these properties c4da neurons are an ideal model system to answer fundamental neurobiological questions and to study the specific impact of disease-related genes on neuronal development, function and maintenance [(28–33)](https://sciwheel.com/work/citation?ids=11890859,94576,362890,605347,605615,4456176&pre=&pre=&pre=&pre=&pre=&pre=&suf=&suf=&suf=&suf=&suf=&suf=&sa=0,0,0,0,0,0&dbf=0&dbf=0&dbf=0&dbf=0&dbf=0&dbf=0). At the same time, they are the larval primary nociceptors [(34)](https://sciwheel.com/work/citation?ids=17738198&pre=&suf=&sa=0&dbf=0). Thus, they functionally resemble neurons whose function is impaired in humans suffering from genetic mutations leading to defects in the ceramide *de novo* synthesis pathway [(10,35)](https://sciwheel.com/work/citation?ids=17604503,14503386&pre=&pre=&suf=&suf=&sa=0,0&dbf=0&dbf=0)”

3. What’s the phenotype of CerSP61 allele? In Fig2 and Fig3, it would help to provide its dendritic morphology, so as to complement the lipidomic analysis of this mutant.

We thank the reviewer for this suggestion. We now analyzed the dendritic morphology of *cerS^P61^* mutants and found that also in this allele dendritic morphology of c4da neurons in simplified. We added those results to figure 2 and added the following statement to the text: p. 7, l 225-226: “Although the mRNA reduction is mild enough to allow escapers to develop, it is still sufficient to observe simplified dendrites in c4da neurons of wandering LIII larvae (Fig. 2A).”

4. Figure 4: it is assumed that mammalian CerS4 and CerS2 can restore dendritic morphology in the CerSH215D mutant via producing C18-C24 ceramide. However, lipidomic evidence was missing to show that medium-long chain ceramide was changed.

We agree that doing lipidomics to test all relevant genotypes would be ideal. Unfortunately, *cerS^HD^* mutants do not survive. We elaborated in our manuscript why the *cerS^P61^* allele was used instead of *cerS^HD^* to study the lipidome of a *cerS* deficient animal. P.7,l.221-225: “*cerS^KO^* and *cerS^H215D^* homozygous mutant animals die very early in development as small larvae, which morphologically correspond in size to first instar. To study the lipidome of a full body *cerS* mutant, we have therefore used the weaker *cerS^P61^* allele. *cerS* mRNA is reduced by approximately 60% in this mutant, which allows a low number of slim larvae to reach the LIII stage [(24)](https://sciwheel.com/work/citation?ids=154254&pre=&suf=&sa=0&dbf=0).”

Our lipidomics using *cerS^P61^* animals show that medium-long-chain ceramides (C18-C14-ceramides) were significantly reduced in the mutant. We rearranged our original figure to make that data more visible (Fig 5A) and mentioned this part in greater detail in the text: p. 10, l.298-302:

“Again, we used homozygous *cerS^P61^* mutant larvae. The most abundant ceramides in wild-type *Drosophila* are C20- and C22-ceramides. Additionally, much lower concentrations of C12-, C16-, C18- and C24-ceramides could be detected. Comparing the abundance of ceramides in the control and the *cerS^P61^* mutant, we found that only ceramides with acyl-chains between C18-C24 carbons were reduced in the *cerS^P61^* mutant (Fig. 5A)

This is just an example, but throughout Figures 2 to 4, the genetic experiments were conducted under the assumption that changes of specific sphingolipid species would happen. To evaluate that all genetic manipulations of enzymes were effective, it is advised that lipidomics be performed.

Following this advice, we have added lipidomics analysis of additional genetic conditions to strengthen the conclusions of these experiments.

First, (also requested by Reviewer 2), we have shown by performing a new lipidomics experiment, that UAS-mCerS5 expression enhances C16-ceramides and C16-Cer-PE production in fruit flies. This result is shown in our new Fig 6A and described in the text on p. 11, l.331-340 :“ To confirm that mCerS5 is functional and that it efficiently generates C16-ceramides in fruit flies, we overexpressed *UAS-mCerS5* ubiquitously using *tubulin-Gal4* and measured the ceramide species composition. These animals had significantly more C16-ceramides indicating that the murine enzyme is active and maintains its substrate specificity in the fruit fly. Also, while we did not detect C16-CerPE in wild-type flies, this lipid species was robustly detected in flies overexpressing mCerS5 (Fig. 6A). To test if a shift in ceramide species composition was leading to a reduction in dendrite complexity, we overexpressed UAS-mCerS5 as well as the other UAS-mCerS constructs in c4da neurons. Only the overexpression of mCerS5 but not mCerS1, mCerS4, or mCerS2 led to reduced dendrite branching in c4da neurons, indicating that increased C16-ceramide levels are detrimental to c4da neurons (Fig. 6B-D).”

Second, in addition to Cer and Cer-PE levels, we added a further analysis of HexCer as an alternative metabolite of Ceramide. This additional data is now added to Fig 2D and explained in the text on p.8,l.229-235: “For example, by adding phosphoethanolamine (PE) through Ceramide phosphoethanolamine synthase (CpeS) ceramides are turned into Cer-PE, the major phosphosphingolipid in *Drosophila*. They can also be converted into hexosylceramides by the addition of glucose or galactose via glucosylceramide synthase (GlcT) (Fig. 2C). We thus measured the relative ceramide, Cer-PE, and HexCer levels. Taking the overall low lipid levels of *cerS^P61^* mutant larvae into account we found the relative Cer-PE level to be significantly decreased (Fig. 2D).”

Third, concerning the functionality of *UAS-SK1* and *UAS-SK2*, we now refer more clearly to the published literature, in which lipidomics were performed to show their activity *in vivo (*Yonamine et al., 2011) on p.9, l.273-277: “The *Drosophila* genome encodes two SKs (SK1 and SK2), that display overlapping functions, as both complement a yeast SK mutant [(49)](https://sciwheel.com/work/citation?ids=319920&pre=&suf=&sa=0&dbf=0). They appear to be functionally redundant in flies, since single SK1 or SK2 mutants are viable while SK1 and SK2 double mutants are lethal. Their overexpression reduces Sphingosine and (dh)S levels, while SK2 overexpression additionally enhances (dh)S1P levels [(14,50)](https://sciwheel.com/work/citation?ids=12666318,9416615&pre=&pre=&suf=&suf=&sa=0,0&dbf=0&dbf=0).”

5. In Figures 2 to 4, do the metabolites (C18-C24 ceramide, CerPE, and (dh)S1P) act within the same pathway in CerS mutants? In other words, how do these alterations in sphingolipids influence each other? Is it possible that the lack of CerPE is a result of the reduced levels of C18-C24 ceramide in CerS mutants? I’d recommend double mutant analyses.

In our resubmitted version we tried to clarify how the metabolites are related to each other and describe more precisely that (dh)S is the substrate for the CerS reaction, (dh)Cer its product and how ceramides are turned into Cer-PE to produce complex SL (a scheme can be found in Fig 2C). In the text we mention the relationship in several passages:

p.8, l. 260: “The Spt-complex generates (dh)S, the substrate for the CerS reaction.”

p.11, l. 319-321 :” It is important to note that in this experiment the substrate for the CerS reaction (dh)S and its metabolite (dh)S1P are lowered and at the same time only ceramides with specific acyl chains are provided. “

p.11, l. 354-357:” Our data show that in *cerS* mutant neurons, both the lack of the end-product of the ceramide *de novo* synthesis pathway (Cer-PE) and the accumulation of the CerS substrate derivative (dh)S1P are deleterious for c4da neurons.”

p. 12, l.359-366:” A comparison of *spt* complex mutants (*spt1* or *lace*) with *cerS^HD^* showed that dendrite morphology was more severely impaired in *cerS* mutants than in *spt* mutant neurons. This result was obtained using two different techniques (MARCM and RNAi). The SPT-complex is upstream of CerS in the ceramide *de novo* synthesis pathway and produces LCBs which are converted into (dh)ceramide by CerS. The difference between SPT-complex and CerS mutants in our study may be explained by the fact that *spt*-complex mutants are mostly suffering from a reduction of complex SL. On the other hand, *cerS^HD^* mutants suffer from a reduction in complex SL and increased LCB levels.”

The relationship between (dh)S and (dh)S1P and how those levels are altered in mutant conditions is summarized in Fig 4A and referred to in the text on p.13, l.398-399:” Naturally occurring LCBs can be phosphorylated to form S1P and (dh)S1P and it was unclear which LCB species is most detrimental to neuronal viability.”

Additionally, we performed new experiments and added the recommended double-mutant analysis by knocking-down the expression of genes that act up-/or downstream of CerS in *cerS^H215D^* mutant c4da neurons. These results are included in the new Fig. 4E/F and in the main text on p.9, l.280-287: “We next tested whether blocking the *de novo* formation of (dh)S or (dh)S1P could rescue dendrite loss in *cerS^H215D^* mutant neurons. However, knockdown of *lace* or of *sk2* in *cerS^H215D^* mutant c4da neurons did not improve dendritic morphology. We additionally downregulated the expression of a G-protein coupled receptor (Tre1) that binds phospholipids and was recently suggested to act as a S1P receptor in *Drosophila* [(51,52)](https://sciwheel.com/work/citation?ids=4430057,15982543&pre=&pre=&suf=&suf=&sa=0,0&dbf=0&dbf=0). Again, no rescue could be observed (Fig. 4E/F). By contrast, the expression of Sphingosine-1-phosphate lyase (Sply), which degrades (dh)S1P to a fatty aldehyde and phosphoethanolamine, mildly rescued the dendritic phenotype (Fig. 4C-E).”

Furthermore, we performed additional experiments to overexpress *UAS-sk1* or *UAS-sk2* and clarify the impact of (dh)S1P on c4da neuronal morphology. Those data are now presented in Fig. 4C/D and are explained in the text on p.9, l,277-280: “In c4da neurons, the expression of either of the two SKs in wildtype c4da neurons caused dendrite reduction. Additionally, it strongly enhanced the loss of dendrites in *cerS ^H215D^* deficient cells, suggesting a dose-dependent effect of phosphorylated LCBs on neurites (Fig. 4C-D).” and p.9,l.287-292 : “Taken together, while phosphorylated LCBs have been frequently described as anti-apoptotic factors [(53)](https://sciwheel.com/work/citation?ids=16449958&pre=&suf=&sa=0&dbf=0), our data suggest that increased levels of phosphorylated LCBs negatively affects dendrite morphology in a dose-dependent manner. However, genetic manipulations, that presumably should block LCB production, action, or degradation, only very mildly rescued the dendrite loss of *cerS^H215D^* mutant c4da neurons, if at all, suggesting that additional factors might be at play.”

Minor:

6. Supplemental Figure 1 lacks a negative control of other tissues’ expression to confirm that CerS functions cell-autonomously for dendrite maintenance.

The cell autonomous function of CerS was proven by the MARCM experiments: in these experiments, only single cells are mutant, and the observed phenotype reveals the cell-autonomous requirement of CerS function in these neruons. We additionally expressed wild-type CerS only in the individual mutant da neurons, showing that the cell-autonomous expression of CerS is also sufficient to rescue the phenotype. To make this clearer to our readers we extended the description of MARCM and the rescue in our text (p.6, l.162-167):

“To overcome the previously mentioned lethality we used the MARCM (Mosaic Analysis with a Repressible Cell Marker) technique by which single homozygous mutant cell clones are generated in an otherwise heterozygous animal [(38,39)](https://sciwheel.com/work/citation?ids=153229,4407304&pre=&pre=&suf=&suf=&sa=0,0&dbf=0&dbf=0). Single cell clones express UAS-mCD8GFP and can therefore be easily identified. Using MARCM, we obtained homozygous *cerS^KO^* or homozygous *cerS^H215D^* mutant c4da single neuronal cell clones.”

To eliminate any additional doubts to this solid genetics, we added a control to show that our MARCM experiments are not influenced by a heterozygous *cerS* background. We therefore imaged c4da dendritic morphology in heterozygote *cerS^KO^* mutants (Fig. S1A) and described this result on p. 6, l. 169/170: “Importantly, a heterozygote mutant background did not affect the dendritic morphology of c4da neurons (Fig. S1A).”

and p. 6, l. 174-179: “We next confirmed that the observed phenotype was due to the cell-autonomous loss of CerS activity in the c4da neurons. We therefore re-expressed wild-type CerS in the *cerS^KO^* mutant single cell MARCM clones, which rescued the total dendrite length and the total number of dendrite terminal endpoints. These experiments demonstrate that CerS activity is cell-autonomously required in c4da neurons (Fig. 1B-D and Fig. S1A/B)

To finally eliminate any potential doubt in this context, we now included experiments, in which we knocked down *cerS* expression in glia or the epidermis in the new Supp. Fig. 5A-C. These data are now described in the text p. 8, l. 248-254: “To test whether, beyond its cell-autonomous role in cd4a neurons, CerS expression in non-neuronal cells also contributes to dendrite complexity, we knocked down CerS in the epidermis and glia. Both of these cell types are in physical contact with the c4da neurons [(45,46)](https://sciwheel.com/work/citation?ids=70151,154109&pre=&pre=&suf=&suf=&sa=0,0&dbf=0&dbf=0). However, knocking down *cerS* in glia (using *repo-Gal4*) or epidermal cells (using *A58-Gal4*) left c4da neuronal morphology unaffected. These data contradict the possibility of additional requirement of CerS function in the glia or epidermis on c4da neuronal morphology (Fig S4).”

7. What is the potential mechanism leading to dendrite loss in CerSH215D (Fig 1E/F)? In Sup fig2, the results have excluded apoptosis, but it still have not been addressed why there is a loss in dendrite number at the LIIIw stage in CerSH215D.

While with this work we have performed a genetics *tour de force* to identify the role of individual lipid species in the overall phenotype associated with CerS mutations -a point of great clinical interest-, the precise cellular mechanisms through which the various species affect dendrite organization lay outside of the scope of this study. We list potential mechanisms based on the -still- limited literature and highlight the importance to expand these lines of research in the future. p. 14, l.412-416: “Toxic lipids including the above-mentioned LCBs can induce a variety of signaling pathways, many of which converge in caspase-dependent apoptosis. Our study excluded caspase’s role in early dendrite loss, but numerous non-caspase-mediated signaling pathways including ferroptosis or autophagic cell death could be at play. In future, it will be important to identify the signaling pathways triggered in this specific context.”

8. In Figures 2 and 3, it would be beneficial to validate the results of genetic manipulations by drug inhibition or metabolite supplementation, as the authors have shown in Supplementary Figure 4 that treating C16-C18 ceramides to CerS mutant would rescue the dendritic defects. Such metabolite supplement experiment (treating with CerPE to CerS mutant), or drug inhibition (inhibiting SK1 or SK2), should be done also in figs 2-3. I put this as a minor comment provided the lipidomics be performed to supplement figures 2-4.

We followed the Reviewer’s recommendation, and, in our revised manuscript, we provide lipidomics evidence that murine CerS can maintain their substrate specificity also when expressed in fruit flies (UAS-mCerS5, new Fig. 6A). Moreover, we show that after overexpressing UAS-mCerS we could detect high levels of C16-CerPE indicating that the ceramide species determines the CerPE species sub-composition. For these reasons, we fed precursor metabolites, rather than CerPE.

Along those lines, we now added a new experiment, in which we added C22-Ceramides to our original dataset for completeness, as now shown on in Fig.S6.

9. It would greatly improve the manuscript if human neuronal cell lines were also examined to verify the fly findings and the functional consequence of human CerS mutation.

We agree with the reviewer that it would be interesting to repeat our experiments in a human neuronal cell line. We nonetheless feel that these data would be beyond the scope of the present study.

10. The readability of this text could be improved. For example, the sentence "However, the fact that impairment of the function of enzymes up- or downstream of cerS leads to milder phenotypes than impairment of cerS function itself suggests that the strong phenotype in cerS mutant neurons may be due to additional metabolic problems" is too complicated. Similar issues scattered across the manuscript.

We improved the readability of our text, and it was corrected by a native speaker.

11. Does CerS activity play a role in Dscam's regulation of early developmental patterning of dendrites and axons? Similar to findings by Goyal et al., which showed that the loss of SPT in flies leads to the accumulation and mislocalization of Dscam (Down syndrome cell adhesion molecule), ultimately impairing axonal development in the adult brain [PMID 30778062]. The results in this manuscript indicate that SPT mutations also lead to impaired dendritic morphology, suggesting that the regulation of dendritic structure may be involved in a manner similar to how Dscam is regulated. Perhaps the authors could add some discussion?

We thank the reviewer for this remark and added the following paragraph to our discussion

p.12/13, l.368-380: “Interestingly, not only high but also low levels of LCBs were correlated to morphological defects. For example, a lack of LCB production in SPT-complex mutant *Drosophila* neurons was correlated with protein sorting defects and aggregation of the cell recognition protein Down syndrome cell adhesion molecule (Dscam) [(54)](https://sciwheel.com/work/citation?ids=15509760&pre=&suf=&sa=0&dbf=0). Dscam regulates multiple processes in *Drosophila* neurons such as proper axonal morphology, correct axonal innervation of target regions, and dendritic self-avoidance [(32,54,56)](https://sciwheel.com/work/citation?ids=14682,605615,15509760&pre=&pre=&pre=&suf=&suf=&suf=&sa=0,0,0&dbf=0&dbf=0&dbf=0). The link between SPT-complex activity and morphological defects was investigated using the *Drosophila* adult Mushroom body (MB), a neuropil in the central nervous system [(54,57)](https://sciwheel.com/work/citation?ids=15509760,6499781&pre=&pre=&suf=&suf=&sa=0,0&dbf=0&dbf=0). During development, the absence of SPT-complex-generated sphingolipids in MB neurons led to an aggregation of Dscam. Axon and dendrite specific Dscam isoforms were not properly distributed into their target compartments which resulted in a failure to segregate axonal branches into distinct MB lobes [(54)](https://sciwheel.com/work/citation?ids=15509760&pre=&suf=&sa=0&dbf=0). However, it remains unclear whether the lack of LCBs or a lack of complex SL is causing Dscam-mediated axon sorting defects”

Reviewer 2

Reviewer 2 states: “Overall, this a nice study about neuronal dendritic arborization and the impact of ceramide synthase gene function in this process. The experiments are well executed, and interpretations are largely consistent with results”. We thank the Reviewer for this positive evaluation. In the following, we address each of the points she/ he raised (“there are several points that need to be addressed before the article is published”). We are confident that with our response we can convince the Reviewer that the manuscript is now ready for publication.

1. There is a conundrum arising out of the argument put forth by the authors:

They claim that conversion of accumulated dihydrosphingosine to dihydrosphingosine 1-phosphate in the CerS^H215D^ mutant background is a major contributor to dendritic pathology observed in the mutant. When they express mCerS5 they see minimal rescue of the phenotype. If the mCerS5 is active in the transgenic animal, it should consume the accumulated LCBs in CerS mutant background, and the reduced LCBs should afford good rescue if accumulated LCB and LCBP is indeed responsible for dendritic length and branching phenotype. The authors have to confirm that mCerS5 is indeed active in the transgenic animals. If the enzyme is indeed active, it does not answer the conundrum but at least the authors would have an answer to a caveat that is raised by their results.

We thank the reviewer for this remark and we addressed this point experimentally. We used the ubiquitous driver tubulin-Gal4 to overexpress UAS-mCerS5 and analyzed the impact of this manipulation on ceramide species distribution. Lipidomics revealed that C16-ceramides and Cer-PE are increased in these animals, confirming that mCerS5 produces C16-ceramides in *Drosophila.* We added those data to our new l figure 6 and described them in the text p.11, l. 331-340:” To confirm that mCerS5 is functional and that it efficiently generates C16-ceramides in fruit flies, we overexpressed *UAS-mCerS5* ubiquitously using *tubulin-Gal4* and measured the ceramide species composition. These animals had significantly more C16-ceramides indicating that the murine enzyme is active and maintains its substrate specificity in the fruit fly. Also, while we did not detect C16-CerPE in wild-type flies, this lipid species was robustly detected in flies overexpressing mCerS5 (Fig. 6A). “

We would also like to point out that, while the small rescue obtained by mCerS5 overexpression might appear confusing, it is a major outcome of our work that each of the alterations induced by impairment of CerS function, contributes to the overall phenotype. It is therefore not surprising that mCers5 overexpression does not yield a full phenotypic rescue.

2. Since it is difficult to work with MARCM clone generated tissues, they should ensure that expression of mCERS1 (we think the reviewer means mCerS5?) in a wild-type background is able to increase C16 ceramide which would mean mCERS5 expression in the *cerS* mutant background is indeed capable of decreasing the LCB and LCBPs. If the argument is Cer-PE is the major determinant of dendritic arborization function it is surprising that an SPT null MARCM clone that should have the most severe reduction in Cer-PE is not as defective as *cerS* mutant.

We overexpressed all UAS-mCerS constructs in c4da neurons. Only UAS-mCerS5 led to a reduction in dendrites suggesting that indeed CerS5 generated lipids are deleterious. The results are shown in our new Figure 6 B-D and described on p. 11, l.336-340: ” To test if a shift in ceramide species composition was leading to a reduction in dendrite complexity, we overexpressed UAS-mCerS5 as well as the other UAS-mCerS constructs in c4da neurons. Only the overexpression of mCerS5 but not mCerS1, mCerS4, or mCerS2 led to reduced dendrite branching in c4da neurons, indicating that increased C16-ceramide levels are detrimental to c4da neurons (Fig. 6B-D).

While those results might appear counterintuitive at first, some of the apparent contradictions derive from the fact that the CerS phenotype results from multiple individual alterations that we defined. To clarify this, we now explain better the relationship between the LCBs and ceramides and also how this contributed to the difference between the phenotypes of the *spt-complex* and the *cerS* mutant animals.

In the following paragraph, we now explain better the fact that neurotoxic LCBs are not formed in spt-complex mutant cells but accumulate and cause additional harm in cerS deficient neurons p. 11, l.354-357: ” Our data show that in *cerS* mutant neurons, both the lack of the end-product of the ceramide *de novo* synthesis pathway (Cer-PE) and the accumulation of the CerS substrate derivative (dh)S1P are deleterious for c4da neurons. We have therefore provided evidence that parallel, individual changes in lipid homeostasis have an impact on dendrite complexity in c4da neurons.”

In addition, we elaborated this point on p. 12, l. 359-366: “A comparison of *spt* complex mutants (*spt1* or *lace*) with *cerS^HD^* showed that dendrite morphology was more severely impaired in *cerS* mutants than in *spt* mutant neurons. This result was obtained using two different techniques (MARCM and RNAi). The SPT-complex is upstream of CerS in the ceramide *de novo* synthesis pathway and produces LCBs which are converted into (dh)ceramide by CerS. The difference between SPT-complex and CerS mutants in our study may be explained by the fact that *spt*-complex mutants are mostly suffering from a reduction of complex SL. On the other hand, *cerS^HD^* mutants suffer from a reduction in complex SL and increased LCB levels.”

3. In the study the authors have argued that their *Drosophila* CerS^H215D^ mutant is a model for the study of PME8 a disease caused by mutations in *CerS 1* gene. That gene should have been their first choice from mammalian genes to rescue and study the sphingolipid profile. Yet, while they have used three of the other mouse genes for rescue, they have neither used *CerS 1* for rescue experiments nor discussed the rationale for not using it in the study.

We agree that not using mCerS1 seemed counterintuitive and we apologize for not explaining why the other mouse CerS enzymes were used in our fist submission. Our lipid analysis has shown (former Figure 4A) that Drosophila CerS produces mostly C20- and C22-ceramides and only low levels of C18-ceramides. Also, C16-ceramides are abundant but not produced by the endogenous CerS since their concentration does not decline in the cerSP61 mutant. Ceramides that are produced by the Drosophila CerS (and that are reduced in the mutant) are C20- and C22- ceramides and these are not produced by mCerS1. Their production covers the substrate specificity of mCerS4 and mCerS2 which is why we have chosen those for our rescue experiments. mCerS5 was used in addition because in mice it has been shown that C16-ceramdies are overproduced in mutant conditions and recent reports suggested that these may inhibit neuronal function. We now explain better the ceramide species alterations we found (p. 9/10, l.294-302): “Since genetically reducing LCB levels could not, or only mildly, improve *cerS^H215D^* mutant c4da neurons, we tested if restoring ceramide/Cer-PE levels would rescue more efficiently. To test which ceramides should be used for rescue experiments, we analyzed which ceramide species are produced by the endogenous fruit fly CerS and thus may be missing in *cerS* mutant cells. Again, we used homozygous *cerS^P61^* mutant larvae. The most abundant ceramides in wild-type *Drosophila* are C20- and C22-ceramides. Additionally, much lower concentrations of C12-, C16-, C18- and C24-ceramides could be detected. Comparing the abundance of ceramides in the control and the *cerS^P61^* mutant, we found that only ceramides with acyl-chains between C18-C24 carbons were reduced in the *cerS^P61^* mutant (Fig. 5A).”

and p.11,l.341-344: “In summary, a complete rescue of the mutant dendritic phenotype can only be obtained by decreasing toxic LCBs and simultaneously providing C20-C22 ceramides, the most abundant ceramide species*.* Thus, our data indicate that these ceramide species are key metabolites and essential for the complex dendrite elaboration of *Drosophila* c4da neurons.”

However, to fully respond to the Reviewer’s concerns, we also generated an *UAS-mCerS1* transgene and tested its capacity to rescue c4da neuronal morphology in *cerS* mutant neurons. This construct led to improved rescue in comparison to the C16-ceramide producing mCerS5, but worse outcome than the C20-, C22-ceramide producing enzymes. This is in agreement with our statement, that it is important to supply neurons with C20- or longer ceramide species. We added this result to our new figure 5 C/D and describe it in the text on p. 10/11, l. 327-330:” Expression of mCerS1, which produces mainly C18-ceramides, improved the c4da neurons phenotype in comparison to mCerS5 overexpression. Nonetheless, total dendrite length and number of dendritic terminals of c4da neuronal dendrites were still reduced compared to control cells (Fig. 5C/D).”

4. Reduction of CerPE levels affects dendrite morphology. However, it will be interesting to see whether reduced levels of hexosylceramides/glucosylceramides also affect the dendrite morphology. Glucosylceramide synthase (GlcT) knockdown may answer this question.

We ordered three UAS-*glcT^dsRNA^* lines to answer this question (VDRC Stock Nr. 44912 and 108064 and Bloomington Stock Nr. 67304 (gift from John Vaughen)). Unfortunately, stock Nr. 108064 was not available and could not be shipped. However, using VDRC Stock Nr. 44912 and Bloomington Stock Nr. 67304, we did not observe reduced dendritic branching. We also revisited our lipidomics data and found that relative HexCer levels in cerSP61 mutants were not decreased. Thise data were added on p.7, l. 228-235: “By modifying their head groups ceramides can be turned into complex SL. For example, by adding phosphoethanolamine (PE) through Ceramide phosphoethanolamine synthase (CpeS) ceramides are turned into Cer-PE, the major phosphosphingolipid in *Drosophila*. They can also be converted into hexosylceramides by the addition of glucose or galactose via glucosylceramide synthase (GlcT) (Fig. 2C). We thus measured the relative ceramide, Cer-PE, and HexCer levels. Taking the overall low lipid levels of *cerS^P61^* mutant larvae into account we found the relative Cer-PE level to be significantly decreased (Fig. 2D).”

and p.8, l. 243-246: “The results confirmed the importance of Cer-PE production for c4da neuronal morphology. By contrast, knocking down *glcT*, which catalyzes Hexosylceramide formation, did not affect c4da neuronal morphology*.*”

5. Recently Drosophila G protein coupled receptor Tre1 was shown to be a potential S1P receptor (Jiakun Chen et al 2024), the authors could try to knockdown this receptor and see if c4da dendrite phenotype restores in cerS mutants.

We thank the reviewer for this suggestion and were happy to test how a cell-autonomous knockdown of *tre1* would affect/rescue dendrite morphology in a *cerS^H215D^* mutant c4da neurons. We could not see a rescue and added this result in Fig 4E/F and describe it on p. 9, l.283-285: ”We additionally downregulated the expression of a G-protein coupled receptor (Tre1) that binds phospholipids and was recently suggested to act as a S1P receptor in *Drosophila* [(52,53)](https://sciwheel.com/work/citation?ids=4430057,15982543&pre=&pre=&suf=&suf=&sa=0,0&dbf=0&dbf=0). Again, no rescue could be observed (Fig. 4E/F).”

6. Do you see any reduction in mature c4da neuron count in cerS mutants compared to controls? What are the other cells in that are brightly labelled in Supp Fig.2E (left/right corners).

We addressed this valuable question and investigated if larval *cerS* mutant MARCM clones are surviving until the late pupal stage. Additionally, we knocked down *cerS* and imaged whole larvae and adult abdomen. No loss of c4da neurons could be observed. These results are shown in our new Fig. S3 and mentioned in the text p. 7, l. 199-211: “We next tested if CerS is required for the maintenance of c4da neurons and followed five individual *cerS^KO^* c4da mutant MARCM clones from the third larval instar to the late pupal stage. Although the dendrite morphology of the dorsal *cerS^KO^* mutant MARCM clones was largely affected (Fig. S2F/G) all clones could be detected in larva and late pupa, indicating that these cells do not die during this period. To confirm that there is no premature loss of c4da neurons, we additionally knocked down *cerS* in all c4da neurons, counted their cell number in wandering LIII larvae and 5-7 days old adults and found no difference. (Fig. S2D/E). In contrast to the dorsal c4da neurons, the ventro-lateral v´ada c4da neurons can also be visualized also in adult animals because they lie beneath the more transparent ventral side of the abdomen (Fig. S2) [(42)](https://sciwheel.com/work/citation?ids=605427&pre=&suf=&sa=0&dbf=0). We thus analyzed ventro-lateral v´ada c4da neurons at 5-7 days post-eclosion but found no loss (N = 5 animals) (Fig. S2H). Overall, these data indicate that *cerS* mutant c4da neurons exhibit aberrant dendrite morphology but do not show signs of caspase activation and do not undergo apoptosis.”

There are several issues that can be addressed by editing the manuscript appropriately.

7. Lines 56-57: seems like an incorrect statement The refences cited do not provide data or support the notion that myoclonic seizures appear to be related to loss of CerS activity in glia. It should be noted that CerS1 has been shown to be expressed both in neurons and glia in mammals and the mutations have effects in the whole animals include neurons, glia and the liver.

We thank the reviewer for this remark and we have removed “that appear to be related to loss of CerS activity in glia” from that sentence. Instead we now mention *cerS* expression outside of the nervous system (p. 3 l. 81-83): “However, the broad expression pattern of *cerS* genes complicates the elucidation of their nervous system specific function [(13–16)](https://sciwheel.com/work/citation?ids=13486185,12666318,13353731,13353739&pre=&pre=&pre=&pre=&suf=&suf=&suf=&suf=&sa=0,0,0,0&dbf=0&dbf=0&dbf=0&dbf=0)”

8. Lines 63-64: The reviewers could not find any information indicating that epilepsy and the complex phenotype associated with the heterozygous loss of chromosome 1q21 is classified as PME8. Please provide the proper reference or state this in a separate sentence as a related CerS related disorder.

The reviewer is correct, the reference classifies the deletion 1q21 as PME not PME8. This was corrected (p. 3, l. 81) “….gene also results in PME”

9. Lines 104-105: Cer^H215D^ is not a synonymous mutation to Human Myoclonus Epilepsy 8 patient mutation. The original report in EMBO in 2009 by one of the co-authors generated this allele as an active site mutant. The generation and use of Cer^H215D^ as a PME8 mutation (as the authors claim in the text) is puzzling. The authors have taken the trouble of generating the mutation and using in vivo recombination to generate an allele that is driven from the genomic locus. If the intention of the authors was to study HME8 mutation and its pathogenesis they should have generated Cer^H215Q^ allele instead of CerH215D. While the enzymatic activity is compromised in these patients it is not known if they are true null alleles, whereas the Cer^H215D^ has been shown to behave like null in this and previous study. Therefore, the authors are advised to remove this statement since their mutant does not reproduce the exact molecular replacement found in PME8 patients. Similarly, lines 118 and 119 needs to be modified.

We thank the reviewer for this remark and changed the text passages concerned to

(p. 4, l. 123-125): “Here, we used *Drosophila* class four dendritic arborization (c4da) neurons to study the effects of CerS dysfunction on neurons. The complex dendrites of the c4da neurons are housed directly under the transparent cuticle of the larva which facilitates *in vivo* imaging [(27)](https://sciwheel.com/work/citation?ids=12023346&pre=&suf=&sa=0&dbf=0).

and

(p. 5, l.142-150): “To elucidate the impact of CerS dysfunction in *Drosophila* neurons, we first generated a new *cerS* allele in which the endogenous *cerS* gene was replaced by a mutant variant. To do so we used a previously published *cerS^KO^* allele in which most of the protein coding exons were removed and replaced by an attP landing site (28). We then used this landing site to re-integrate the missing exons encoding the wild-type sequence (*cerS^WT^*) as a control [(36)](https://sciwheel.com/work/citation?ids=7543400&pre=&suf=&sa=0&dbf=0), or a sequence bearing a specific mutation. The mutation we introduced replaces histidine at position 215 with aspartic acid. Histidine^215^ is located within the lag1p motif and thus resembles the human *cerS1^H183Q^* mutation. Additionally, a mutation in histidine to aspartic acid at this position has previously been shown to abolish enzymatic activity (Fig. 1A) [(7,17,36)](https://sciwheel.com/work/citation?ids=5768968,10388499,7543400&pre=&pre=&pre=&suf=&suf=&suf=&sa=0,0,0&dbf=0&dbf=0&dbf=0).”

10. Also, in figure 1A hCerS1^H183C^ should be corrected to hCerS1^H183Q^.

We corrected hCerS1^H183C^ to hCerS1^H183Q^.

11. Lines 315-316: HSAN1 neuropathy is a dominant condition, which means phenotypes are seen in heterozygous patients. PME8 is caused by synonymous or non-synonymous homozygous mutations in CERS1. Homozygous mutations of SPT are lethal in mice and other organisms. Also, it is well established that the mutant forms of the enzymes demonstrate altered substrate specificity and produce deoxysphingolipids from the altered substrate utilization (alanine and glycine) that could be toxic. It is advised the authors edit lines 306-316.

We agree with the reviewer that this should be clarified. We changed the text my explaining better the nature of SPT mutations that lead to the formation of the atypical 1-deoxisphingoloipids in contrast to those alleles that enhance the production of typical LCBs p. 12/13, l. 384-397: “HSAN1 is caused by alterations in the SPT-complex that acts upstream of CerS in the ceramide *de novo* synthesis pathway. So far, 17 point mutations have been mapped. The best-studied point mutations change the substrate specificity of the SPT complex. This complex typically condensates serine with acyl-CoA but these mutations allow it to efficiently utilize L-alanine and glycine which then forms atypical LCBs. Such 1-deoxysphinganine and 1-(deoxymethyl)sphinganine lack the hydroxy group which serine would provide. The serine-derived hydroxy group in typical LCBs is critical for a conversion into complex SL and also for degradation. Thus, its lack prevents 1-deoxisphingoloipids not only from being converted into complex SLs and also to be degraded through the S1P-Lyase pathway [(10,11,35)](https://sciwheel.com/work/citation?ids=14503386,16487238,17604503&pre=&pre=&pre=&suf=&suf=&suf=&sa=0,0,0&dbf=0&dbf=0&dbf=0). Of greater interest to our study, three of the 17 above-mentioned mutations increase the enzymatic activity. This results in an increase in common LCBs, such as (dh)Sphingosine. Patients carrying these mutations show exceptionally severe HSAN1 symptoms. Therefore, high plasma LCB levels were suggested as a biomarker that could help to predict the severity of the HSAN1 [(10)](https://sciwheel.com/work/citation?ids=14503386&pre=&suf=&sa=0&dbf=0).”

12. Line 235: Please edit ‘out’ to our

13. Line 313: Please edit ‘spingoid to sphingoid

14. Line 322: Please edit ‘trough’ to through

We corrected those typos.
