# Supplementary material for: Individual lipid alterations at the origin of neuronal Ceramide Synthase defects
Source: PLoS Genet. 2025 Sep 25;21(9):e1011880. doi: 10.1371/journal.pgen.1011880 (PMC12500085; doi:10.1371/journal.pgen.1011880)
Supplement: Attachment — Submitted filename: Comments_to_the_Authors_II.docx [file pgen.1011880.s011.docx]

**Comments to the Authors:
Please note here if the review is uploaded as an attachment.**

Reviewer #1: The authors have significantly improved the manuscript. I have no further questions. Recommended.

Reviewer #2: The authors have addressed all the concerns satisfactorily. It is a delight to recommend the manuscript for publication in PLoS Genetics.

We thank Reviewer 1 and Reviewer 2 for the positive evaluation of our revised manuscript and are happy that both recommend our manuscript for publication in PLoS Genetics.

Reviewer #3: The authors of this manuscript aimed to investigate the effects of disturbed lipid metabolism on the brain. To do so, they used Drosophila larvae that had specific enzymes involved in the ceramide synthesis pathway inactivated. To relate the resulting changes to Progressive Myoclonic Epilepsy Type 8 (PME8), they primarily used the morphology of Drosophila class four dendritic arborization (c4da) neurons as their metric and also used lipidomics to determine the levels of several relevant ceramide species. They found that loss of function of the Drosophila CerS enzyme not only resulted in the reduction in the levels of specific ceramide species but also neuronal degeneration. In their attempt to identify a potential therapeutic avenue, they discovered that full rescue of neuronal morphology could not only be achieved by supplementation of the missing ceramides but also requires removing the buildup of toxic CerS substrates.

We would like to thank the Reviewer for critically reviewing our revised manuscript and are happy to address all the points he raised while reading this revised version

Major Comments
1. Page 7, Lines 202 – 209: The order of the sub-figures does not make sense. Dendrite morphology comes first in the text but is labelled as S2F/G. The confocal images of LIII come after that in the text, but are labelled S2D/E.

We agree with the reviewer, and we have changed the order of the subfigures in Fig. S2. We now present the pupal MARCM clones first (previous Fig. S2F/G now Fig. S2D/E) and changed the text accordingly (p. 7, l. 198-203): ”We next tested if CerS is required for the maintenance of c4da neurons and followed five individual *cerS^KO^* c4da mutant MARCM clones from the third larval instar to the late pupal stage. Although the dendrite morphology of the dorsal *cerS^KO^* mutant MARCM clones was largely affected (Fig. S2D/E) all clones could be detected in larva and late pupa, indicating that these cells do not die during this period.”

2. A comparison between LIII larvae and adults is discussed on line 205, but S2D/E only shows LIII larvae. The paragraph then finishes with the missing adults, but this is labelled S2H when it should logically follow the previous sub-figure. This is confusing and the figures should be re-ordered.

We agree that this was confusing and changed the order of the subfigures. The previous Fig. SD/E became Fig. S2F/G (p. 7, l. 203-206): “To confirm that there is no premature loss of c4da neurons, we additionally knocked down *cerS* in all c4da neurons and investigated if all c4da neurons were present in wandering LIII larvae. Indeed, at this stage all dorsal ddaC (Fig. S2F), ventro-lateral v´ada, and ventral vdaB (Fig. S2G) c4da neurons could be imaged.”

3. Dorsal images of LIII larvae does not seem useful, given that these cannot be compared to the adults, which was the point being made.

We performed this experiment in response to Reviewer 2, who was wondering if there is a premature loss of mutant c4da neurons. We would like to keep the dorsal and ventral images to show that we could not observe any loss of larval c4da neurons after *cerS* knock-down in all c4da neurons. However, we have modified the text to better explain why we look at all three c4da neuronal subtypes in larvae but only at one c4da neuronal subtype in adult flies (p. 7, l. 203-214):

“To confirm that there is no premature loss of c4da neurons, we additionally knocked down cerS in c4da neurons and investigated if all c4da neurons were present in wandering LIII larvae. Indeed, at this stage all dorsal ddaC (Fig. S2F), ventro-lateral v´ada, and ventral vdaB (Fig. S2G) c4da neurons could be imaged. To then ask whether c4da neurons also survive until the adult stage we only imaged the abdominal ventro-lateral v´ada c4da neurons. Those, in contrast to other c4da neurons, such as the ddaC neurons, can be visualized also in adult animals because they lie beneath the more transparent ventral side of the abdomen. Additionally, and in contrast to vdaB neurons, they do not undergo age-dependent apoptosis during the pupal stage [(42)](https://sciwheel.com/work/citation?ids=605427&pre=&suf=&sa=0&dbf=0). Here, all v´ada c4da neurons were present at 5-7 days post-eclosion (N = 5 animals) (Fig. S2H). Overall, these data indicate that *cerS* mutant c4da neurons exhibit aberrant dendrite morphology but do not show signs of caspase activation and the lack of CerS function does not influence c4da neuronal viability.”

Minor Comments
4. Page 3, Line 87: The lag1p “motif” is misspelled as “motive”. Please correct it

This has been corrected

5. Page 5, Line 131 – 133: Both cited papers (10,35) discuss Hereditary Sensory Neuropathy Type I, which is most commonly caused by a mutation in the SPT1 gene, which catalyzes the initial step in sphingolipid biogenesis. While the nociceptors are likely to be affected in that disorder, it seems like a stretch to say that any genetic mutation affecting the ceramide synthesis pathway will also affect the nociceptors. This claim needs to be better supported by literature exploring other disorders of ceramide biogenesis (such as those mentioned at the start of the introduction).

We agree with the reviewer that we should be more precise. In this paragraph we mention diseases caused by problems in the ceramide *de novo* synthesis pathway. The diseases mentioned before in the introduction are related to problems in ceramide *de novo* synthesis or SL degradation. We modified the original sentence to match with the references: p.5, l. 131-133 “Thus, they functionally resemble neurons whose function is impaired in humans suffering from HSAN1. HSAN1 is caused by mutations upstream of CerS which also affect the ceramide *de novo* synthesis pathway [(10,35)](https://sciwheel.com/work/citation?ids=17604503,14503386&pre=&pre=&suf=&suf=&sa=0,0&dbf=0&dbf=0).”

6. Page 6, Line 186: The figure reference was likely meant to be “Fig. S1D” and not C. Please double check.

This has been corrected

7. Page 6, Line 190 – 192 and Figure 1E/F: The degeneration was described as “progressive loss” but there was only a single time point studied (24 hours after the control). Without more time points in between the control and the +24 hour period, it would be impossible to say if the degeneration occurred progressively over the hours or suddenly just before the 24 hour mark. Unless you have data showing which one it might be, it might be best to simply describe it as a “loss”.

We agree with the reviewer and we have removed “progressively” in this paragraph p.6, l.190-193: “To specifically address the loss of dendrite branchlets during the LIII stage, we imaged *cerS* deficient MARCM clones at the early LIIIf stage and re-imaged the same cells 24h later. This analysis confirmed the loss of identifiable dendrites in *cerS^H215D^* mutant c4da neurons (Fig. 1E/F) or in *cerS^KO^* mutant c4da neurons (Fig. S1E).“

8. Supplemental Figure S1E: This figure was not cited in the text.

This has been corrected

9. Page 7, Lines 211 – 216: Please explain why a knockdown model was used when there are mutants available that are used elsewhere in the study.

We thank the reviewer for this comment and now explain why a KD model is used for this experiment p.7, l. 217-219: “To ensure the reduction of CerS function in all c4da neurons, we knocked down *cerS* via RNAi instead of using MARCM, which yields only a very small number of mutant da neurons per animal.“

10. Page 7, Lines 213 – 214: From Figure 1G, there appears to be no significant difference between the middle group and the control. However, the text reads as though both groups were significantly different. Which is the case?

We agree with the reviewer that our way of showing the significance levels in this subfigure was confusing. This has been corrected in Fig 1G and the text on p. 7, l. 219-224: “Animals harboring the *UAS-cerS^dsRNA1^* construct alone (*UAS-cerS^dsRNA1^*/+) showed already a slight impairment in their mechano-nociceptive response compared to animals of the second control genotype (*21-7-Gal4 > UAS-Dicer2/+)* and merely a slight non-significant reduction of their nociceptive responses. However, the *UAS-cerS^dsRNA2^* resulted in significantly fewer nociceptive responses compared to both control genotypes (Fig. 1G), suggesting that *cerS* is also required to maintain c4da neuron function.”

11. Figure 2D: The HexCer graph is missing its units on the Y-axis

This has been corrected

12. Page 10, Lines 302 – 304 and Page 14, Lines 447 - 449: Given that the larvae were raised identically (presumably), would that not rule out exogenous sources? What would you say would be the likely source of difference between the two?

We tried to clarify that point by modifying the text on (p. 10, l. 308-313) to make clear that only ceramide species, that decrease in the mutant are most likely produced by the fly CerS. Ceramides that do not decrease must be taken up from the diet or produced and provided by gut bacteria:

“Comparing the abundance of ceramides in the control and the *cerS^P61^* mutant, we found that only ceramides with acyl-chains between C18-C24 carbons, but not C12- or C14-ceramides, were reduced in the *cerS^P61^* mutant (Fig. 5A). This suggests that Drosophila CerS produces ceramides with C18-C24 acyl chains and that C12- or C16-ceramides, which are found in robust amounts in mutants and controls, may originate from exogenous sources such as food or gut bacteria.”

13. Page 10, Lines 308 – 312: In Figure S5B, it seems only C22 had a significant effect, but the text implies C18 was also significant. Which was the case? There also should be normal control to better understand how mild the improvement is in comparison to normal larvae.

We changed the text on p.10, l. 320-321 to: “Indeed, supplementation with C22-ceramides led to a mild improvement of c4da dendrite complexity (Fig S5).” and added the CerS^WT^ control to figure S5 “

14. Table S2: *** is repeated twice. The ‘<’ sign is probably missing from the last two p-values. Please double check that.

This has been corrected

15. Page 13, Line 405: The correct figure is probably Fig 4. Please double check.

This has been corrected
